# *Geminicoccus flavidas* sp. nov. and *Geminicoccus harenae* sp. nov., two IAA-producing novel rare bacterial species inhabiting desert biological soil crusts

**DOI:** 10.3389/fmicb.2022.1034816

**Published:** 2022-10-31

**Authors:** Zhu-Ming Jiang, Yang Deng, Xue-Fei Han, Jing Su, Hao Wang, Li-Yan Yu, Yu-Qin Zhang

**Affiliations:** ^1^Institute of Medicinal Biotechnology, Chinese Academy of Medical Sciences, Beijing, China; ^2^State Key Laboratory of Dao-di Herb, Beijing, China

**Keywords:** *Geminicoccus flavidas*, *Geminicoccus harenae*, average nucleotide identity, pan-genome, biological soil crusts

## Abstract

Two Gram-staining negative strains (CPCC 101082^T^ and CPCC 101083^T^) were isolated from biological sandy soil crusts samples collected from Badain Jaran desert, China. Both isolates were heterotrophic phototroph, could produce indole-3-acetic acid. The 16S rRNA gene sequences of these two strains were closely related to the members of the family *Geminicoccaceae*, showing high similarities with *Geminicoccus roseus* DSM 18922^T^ (96.9%) and *Arboricoccus pini* B29T1^T^ (90.1%), respectively. In phylogenetic tree based on 16S rRNA gene sequences, strain CPCC 101082^T^ and CPCC 101083^T^ formed a robust distinct clade with *Geminicoccus roseus* DSM 18922^T^ within the family *Geminicoccaceae*, which indicated that these two isolates could be classified into the genus *Geminicoccus*. The growth of strain CPCC 101082^T^ occurred at 15–42°C and pH 4.0–10.0 (optima at 28–37°C and pH 6.0–8.0). The growth of strain CPCC 101083^T^ occurred at 4–45°C and pH 4.0–10.0 (optima at 25–30°C and pH 6.0–8.0). The major cellular fatty acids of CPCC 101082^T^ and CPCC 101083^T^ contained C_18:1_*ω*7*c*/C_18:1_*ω*6*c*, cyclo-C_19:0_*ω*8*c*, and C_16:0_. Q-10 was detected as the sole respiratory quinone. Diphosphatidylglycerol, phosphatidylglycerol, phosphatidylcholine, phosphatidylethanolamine, an unidentified phospholipid and an unidentified aminolipid were tested in the polar lipids profile. The genomes of the two isolates were characterized as about 5.9 Mbp in size with the G + C content of nearly 68%. The IAA-producing encoding genes were predicated in both genomes. The values of average nucleotide identity were 80.6, 81.2 and 92.4% based on a pairwise comparison of the genomes of strains CPCC 101082^T^ and CPCC 101083^T^ and *Geminicoccus roseus* DSM 18922^T^, respectively. On the basis of the genotypic, chemotaxonomic and phenotypic characteristics, the strains CPCC 101082^T^ (=NBRC 113513^T^ = KCTC 62853^T^) and CPCC 101083^T^ (=NBRC 113514^T^ = KCTC 62854^T^) are proposed to represent two novel species of the genus *Geminicoccus* with the names *Geminicoccus flavidas* sp. nov. and *Geminicoccus harenae* sp. nov.

## Highlights

This is the first report about the rare bacterial genus *Geminicoccus* was obtained from the desert niches. The primary goal of the present research was to identify the *Geminiocccus* cultures from Badain Jaran Desert, and to study the properties of *Geminicoccus* members inhabiting desert niches. The detailed phenotypic and genotypic properties resulted from these strains could be helpful to systematically demonstrate the ecological adaptation mechanism and their ecological function of the rare bacteria. As a result, strains CPCC 101082^T^ and CPCC 101083^T^, isolated from sandy soil samples collected from Badain Jaran Desert, showed indole-3-acetic acid (IAA)-producing activity. Based on the phenotypic and genotypic data, two novel species *Geminicoccus flavidas* sp. nov. and *Geminicoccus harenae* sp. nov., are proposed, with the isolates CPCC 101082^T^ and CPCC 101083^T^ as the type strains, respectively.

## Introduction

The genus *Geminicoccus* was initially proposed by Foesel et al. (2007), with the type species *Geminicoccus roseus* (type strain DSM 18922^T^) as the sole species. Originally, the genus *Geminicoccus* was classified in the order *Rhodospirillales* without being designed into any family. As of 2008, based on the core genes analysis on the available genomes of the members in the order *Rhodospirillales* and the newly isolated strain B29T1^T^, which was isolated from the endophytic microbial community of a *Pinus pinaster* treetrunk, Proença et al. established the family *Geminicoccaceae*, with the genus *Geminicoccus* as the type genus, and also proposed a new genus *Arboricoccus*, with strain B29T1^T^ being the type strain of the type species *Arboricoccus pini* (Proenca et al., 2018). There are currently only four recognized genera: *Arboricoccus*, *Defluviicoccus*, *Geminicoccus*, and *Tistrella* being classified in the family *Geminicoccaceae*^1^ and there is one species in each genus. To summarize, currently, only strain DSM 18922^T^, isolated from a marine aquaculture biofilter, is accommodated in the genus *Geminicoccus*. Cells of the genus *Geminicoccus* are diplococci, non-motile, aerobic and heterotrophic phototroph. The present study aimed to establish the taxonomic status of two newly isolated *Geminicoccus* strains (CPCC 101082^T^ and CPCC 101083^T^) recovered from desert biological soil crusts samples collected from Badain Jaran desert, China. The isolates were pairwise compared with one another and as well with the type strain DSM 18922^T^ of the currently described sole *Geminicoccus* species, and further studied using a polyphasic taxonomic approach. The isolates, strains CPCC 101082^T^ and CPCC 101083^T^, were found to represent two new *Geminicoccus* species, respectively, for which the respective names *Geminicoccus flavidas* sp. nov and *Geminicoccus harenae* sp. nov. are proposed.

## Materials and methods

### Acquisition of samples

The sandy soil samples were collected from Badain Jaran desert in Alxa Right Banner, Alxa League, China. Six samples sandy soil samples were collected at random from a hinterland area (39°34′24″N, 101°47′56″E, 1286 mH) characterized by cyanobacteria-dominated crusts, pooled and marked as BJDC. Another sandy soil mixture labeled as BJDM was obtained around a sand dune (39°18′45″N, 101°55′37″E, 1580 mH) with moss-dominated crusts. These samples were separately sealed in sterile envelopes and sent to the laboratory within 1 week after collection. All samples were immediately processed for isolation of microorganisms after arriving at the laboratory, and the remaining samples were maintained at −80°C.

### Isolation and identification of bacterial strains

After dilution of the sample using 0.85% (w/v) NaCl solution mixed with 0.1% sodium pyrophosphate, 200 μl of 10^−4^ concentration soil-suspension was spread onto the isolation medium Difco™ Marine Agar 2,216, which contained (L^−1^): 19.45 g NaCl, 8.8 g MgCl_2_, 5 g peptone, 3.24 g Na_2_SO_4_, 1.8 g CaCl_2_, 1 g yeast extract, 0.55 g KCl, 0.16 g NaHCO_4_, 0.1 g ferric citrate, 0.08 g KBr, 34 mg SrCl_2_, 22 mg Boric acid, 8 mg disodium phosphate, 4 mg sodium silicate, 2.4 mg NaF, 1.6 mg NH_4_NO_3_, 15 g agar; pH 7.6–7.8. Nystatin (50 mg L^−1^) and potassium dichromate (30 mg L^−1^; Shi et al., 2019) were added to the isolation medium to prevent the growth of fungi that might be present. After 4 weeks of incubation at 28°C, distinct colonies were picked and streaked onto PYG plates (5.0 g L^−1^ yeast extract, 3.0 g L^−1^ peptone, 10.0 ml L^−1^ glycerol, 1.25 g L^−1^ betaine hydrochloride, 1.25 g L^−1^ sodium pyruvate, 15 g L^−1^ agar) until the isolated and uniform colonies appeared. The purified culture was temporarily maintained on GYM agar (4 g glucose, 4 g yeast extract, 10 g malt extract, 2 g CaCO_3_, 15 g agar, pH 7.0–7.2) at 4°C and stored as aqueous glycerol suspension (20%, v/v) at −80°C.

The reference strain of *G. roseus* DSM 18922^T^ obtained from DSMZ was included in parts of the following assays as a parallel.

The newly isolated bacterial strains were primarily identified according to the 16S rRNA gene sequence comparison in the following steps.

Extraction of the genomic DNA of strains CPCC 101082^T^ and CPCC 101083^T^ and sequencing of PCR amplification products of 16S rRNA genes were performed as described by Li et al. (2007). The obtained sequences were compared with available 16S rRNA gene sequences from GenBank using the BLAST program and EzBioCloud^2^ to determine the approximate phylogenetic affiliations of the strains (Kim et al., 2012). Multiple alignments with sequences of the most closely related taxa and calculations of levels of sequence similarities were carried out using MEGA version 11.0 (Tamura et al., 2021). Phylogenetic trees were inferred using the neighbor-joining method (Saitou and Nei, 1987) with K_nuc_ values (Kimura, 1979; Kimura, 1980) and complete deletion gaps, and the maximum-parsimony (Kluge and Farris, 1969) and maximum-likelihood (Felsenstein, 1981) methods. The topology of the phylogenetic tree was evaluated by the bootstrap resampling method of Felsenstein (Felsenstein, 1985) with 1,000 replicates.

### Observation of morphological properties and physiological tests

Cell Gram-staining was performed by standard Gram’s reaction. Cell morphology was observed by a light microscope and confirmed using transmission electron microscopy (JEM-1010, JEOL). The mobility of the cells was checked by inoculating cells into GYM medium (g L^−1^; glucose 4, yeast extract 4, malt extract 10, calcium carbonate 2, pH 7.5) with 0.3% (w/v) agar and incubated at 30°C for 5 days.

The growth conditions of the isolates were tested using GYM, Tryptic soy agar (TSA, Difco), Reasoner’s 2A agar (R2A, Difco), nutrition agar (NA) and PYG agar by incubating the cultures for up to 7 days. The temperature range of cell growth was tested at 4, 10, 15, 20, 25, 28, 30, 32, 35, 37, 40, and 45°C using GYM slant medium in multi-thermo incubators (Eyela, MTI-20; Tokyo Rikakikai). The growth of the strains at different pH values (pH 4–12, at intervals of 1 pH unit) and NaCl concentrations (0–15%, w/v, at intervals of 1%) was examined using GYM broth at 30°C. Growth under anaerobic conditions was determined on GYM slant medium for 7 days at 30°C in an anaerobic incubator. Growth under light and dark conditions was monitored on GYM agar medium for 7 days at 30°C in an illumination incubator and ordinary constant temperature incubator, respectively. Cells were collected by centrifugation. About 0.5 g of the pellets were resuspended in 15 ml saline solution then centrifuged to remove the remained medium. Pigments were extracted by chloroform and analysed by HPLC (Reddy et al., 2006) and absorption spectra between 190 and 900 nm were recorded in a UV/Vis spectrophotometer (TU-1901, PERSEE; Kämpfer et al., 2003).

Catalase activity was evaluated by bubble production in 3% (v/v) H_2_O_2_, and oxidase activity was assessed by API oxidase reagent (bioMeriéux). Metabolic characteristics were examined using Biolog GEN III (MicroPlate), API 50CH (bioMerieux), API 20NE (bioMerieux) and API ZYM test kits (bioMerieux) according to the manufacturer’s instructions. H_2_S production and nitrate reduction were tested according to previously described procedures (Dong et al., 2014). Endoglucanase activity was examined using CMC-Na screening medium (Reinhold-Hurek et al., 1993). The abilities to hydrolyse gelatin and starch and other physiological tests were carried out as described by Gonzalez et al. (1978). Voges-Proskauer (VP) and Methyl-Red (MR) tests were performed on glucose–peptone broth medium. The ability of the strains to produce IAA was tested using colorimetric methods (Bric et al., 1991). Cells in the logarithmic phase of growth were inoculated into 1% tryptone aqueous solution containing 3 mmol/L L-tryptophan and then cultured at 30°C for 3 days. The concentration of IAA standard solution (0, 0.675, 1.25, 2.5, 5, 10, 20, 40, 50, 60, 80, 100 mg/L) was taken as the abscissa and the absorbance value of the corresponding concentration at 540 nm as the ordinate to draw a scatter diagram, and a linear trend line was added to obtain the IAA standard curve, as shown in Supplementary Figure S1.

### Chemotaxonomic tests

Cells for chemotaxonomic and molecular systematic studies were collected from GYM broth at 30°C for 5–7 days, except the cells for fatty acids analysis. The polar lipids were extracted and isolated by two-dimensional TLC and identified by previously described procedures (Minnikin et al., 1984). Ubiquinone(s) was extracted and purified according to the method described previously and analyzed by HPLC (Collins et al., 1977). Cultures for cellular fatty acids extraction were harvested by using TSB broth (Difco) at 30°C for 5 days. Analysis of the whole-cell fatty acid profile was carried out by the Sherlock Microbial Identification System (MIDI) according to the manufacturer’s instructions (Kroppenstedt, 1985).

### Whole-genome study

#### Genome sequencing, assembly and quality evaluation

Genome sequencing was performed on an Illumina HiSeq 4,000 system (Illumina) at BGI company (Shenzhen, China). Genomic DNA was sheared randomly to construct three read libraries with lengths of 300 bp by a Bioruptor ultrasonicator (Diagenode, Denville, NJ, United States) and physico-chemical methods. The paired-end fragment libraries were sequenced according to the Illumina HiSeq 4,000 system’s protocol. Raw reads of low quality from paired-end sequencing (those with consecutive bases covered by fewer than five reads) were discarded. The sequenced reads were assembled using SOAPdenovo v1.05 software. The completeness and contamination of the genomes were accurately estimated by the CheckM pipeline.

#### Genomic component prediction and gene annotation

Gene prediction was performed on the genome assembly by glimmer3^3^ with Hidden Markov models. The tRNAscan-SE (Lowe and Eddy, 1997), RNAmmer and the Rfam databases were employed for sorting tRNA, rRNA and sRNAs, respectively. The annotation of tandem repeats was conducted using the Tandem Repeat Finder,^4^ and the minisatellite DNA and microsatellite DNA were selected based on the number and length of repeat units. The Genomic Island Suite of Tools (GIST) was used for genomic islands analysis^5^ with the IslandPath-DIOMB, SIGI-HMM and IslandPicker methods. Prophage regions were predicted using the PHAge Search Tool (PHAST) web server,^6^ and CRISPR identification was conducted using CRISPRFinder.

The best hit was abstracted using the Blast alignment tool for function annotation. Seven databases, KEGG (Kyoto Encyclopedia of Genes and Genomes), COG (Clusters of Orthologous Groups), NR (Non-Redundant Protein Database databases), Swiss-Prot, GO (Gene Ontology), TrEMBL and EggNOG, were used for general function annotation.

The protein sequences of each genome annotated by the abovementioned methods were used for downstream analysis such as stress-response gene retrieval and pan-genome analysis. For pathway analysis, the predicted protein sequences were uploaded to the KEGG Automatic Annotation Server using the “for prokaryotes” and “bidirectional best hit” options. The UniProt^7^ and Interpro^8^ websites were used for validation of the stress response genes. Predictions of gene clusters for natural products were performed using antiSMASH (Blin et al., 2019).

#### Whole genome-based taxonomy

The draft assembly of the genomes was submitted to GenBank to be publicly accessible. The draft assembly genomic sequence (accession number: ATYL00000000) of the strain *Geminicoccus roseus* DSM 18922^T^ was downloaded from the NCBI database. The genomic G + C content was calculated from the draft genome sequences. Genomic robust indexes, i.e., ANI (average nucleotide identity) and dDDH (digital DNA–DNA hybridisation) were calculated for the definition and classification of the species. The ANI Calculator^9^ (Yoon et al., 2017) and Genome-to-Genome Distance Calculator^10^ (Meier-Kolthoff et al., 2013) were used to calculate the ANI and dDDH, respectively.

The Bacterial Pan-genome Analysis (BPGA) pipeline was applied for analysis of the genomic diversity of the *Geminicoccus* members. The protein sequences of the strains CPCC 101082^T^ and CPCC 101083^T^ were predicted by glimmer 3.02, and the protein sequence of the strain *G. roseus* DSM 18922^T^ was annotated by RAST 2.0. Pan-genome analysis was performed by BPGA 1.3 using default settings (Chaudhari et al., 2016). A total of three protein sequence files annotated from the three corresponding strains’ whole genome sequences were used to generate orthologous gene/protein clusters (homologous families) using the USEARCH clustering tool and to construct the phylogenetic tree using the concatenated core genes using BPGA.

Each homologous family was given a conserved value based on the frequency in the three genomes. Here, we defined the homologous gene families conservation value (CV) as 3, when a certain homologous gene was common shared by all of the type strains of these three species. Different CVs reflect the distribution frequency of the homologous gene among the three strains. A higher CV indicates that the gene is more widely distributed in the strains of the genus *Geminicoccus* and the gene family is more conserved in the genus *Geminicoccus*. In the pan-genome of the three strains, if the CVs of some homologous families were 3, these homologous families were considered part of the core genome; homologous families with CVs of 2 or 1 were regarded as accessory genes or unique genes, respectively. The core, accessory, unique and exclusively absent genes were retrieved using the USEARCH clustering tool. BPGA was used to perform the evolutionary analysis based on concatenated core gene alignments and the binary (presence/absence) pan-matrix. The gene matrix was calculated using similarity or dissimilarity in the contribution of genes to orthologous gene clusters. For the core genome-based phylogenetic tree, BPGA first extracted the protein sequences (excluding paralogs) from 20 random orthologous gene clusters to generate the core genome phylogeny tree. BPGA automated multiple sequence alignments using MUSCLE. All alignments were concatenated, and a neighbor-joining phylogenetic tree was constructed.

## Results and analysis

### Isolation and primary taxonomic study results on *Geminicoccus* strains

Strains CPCC 101082^T^ and CPCC 101083^T^ were recovered from sandy soil samples BJDC and BJDM, respectively.

**Figure 1:**
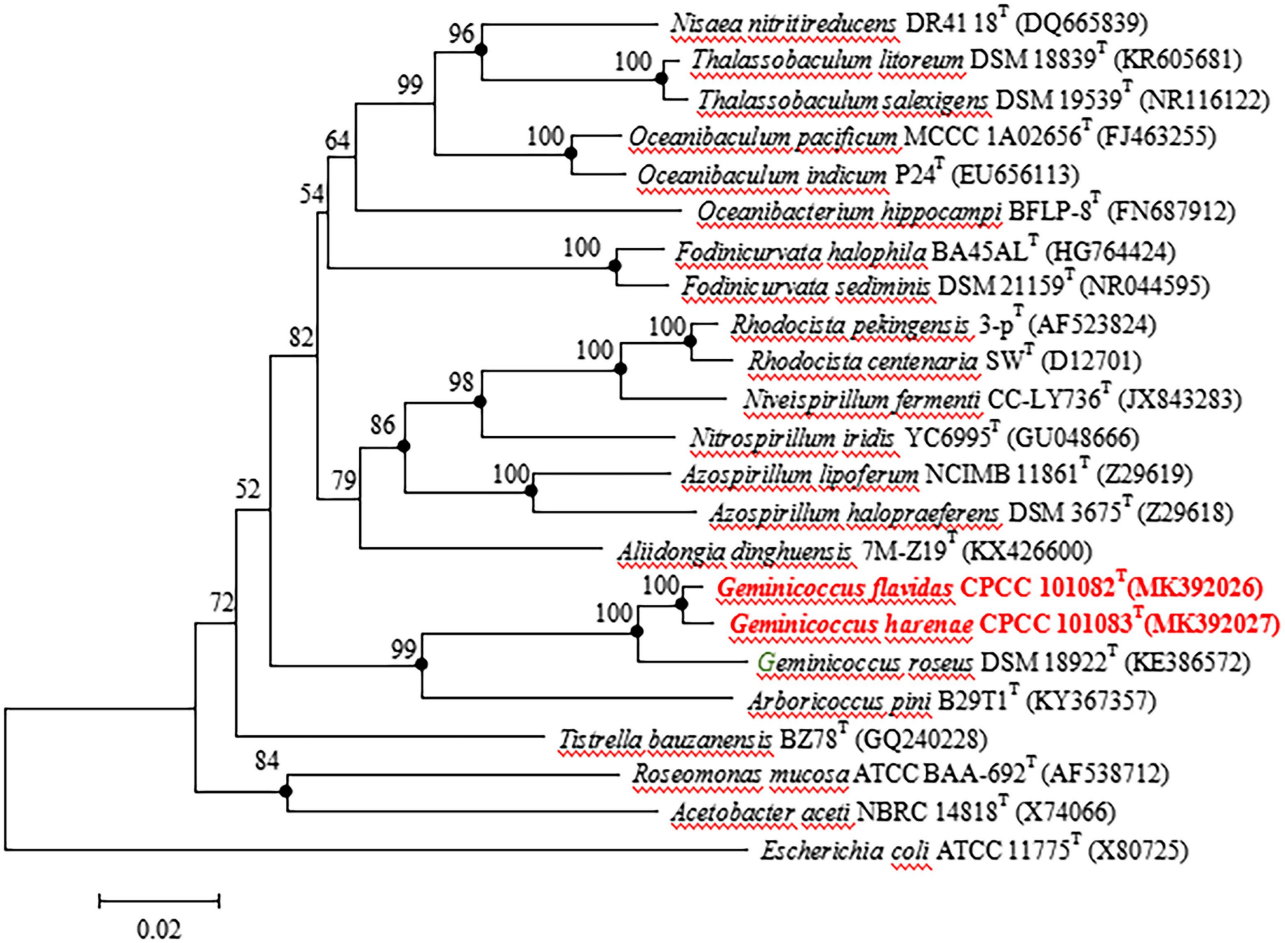
Neighbour-joining tree based on 16S rRNA gene sequences showing the relationship of the strains CPCC 101082^T^, CPCC 101083^T^ and *Geminicoccus roseus* DSM 18922^T^ with representatives of the order *Rhodospirillales*. Filled circles indicate that the corresponding nodes were also recovered in the trees generated with the maximum-likelihood and maximum-parsimony methods. Bootstrap values (>50 %) are shown as percentages of 1000 replicates. Bar, 2 nt substitutions per 100 nt.

The almost full-length 16S rRNA gene sequences (1,449 and 1,453 bp) of strains CPCC 101082^T^ and CPCC 101083^T^ were obtained. The comparison of the 16S rRNA gene sequences indicated that strains CPCC 101082^T^ and CPCC 101083^T^ were members of the family *Geminicoccaceae*, with the highest similarities to *G. roseus* DSM 18922^T^ (CPCC 101082^T^: 96.9%; CPCC 101083^T^: 96.9%) and *Arboricoccus pini* B29T1^T^ (CPCC 101082^T^: 90.8%; CPCC 101083^T^: 90.1%). The similarity of the 16S rRNA gene sequences between CPCC 101082^T^ and CPCC 101083^T^ was 98.6%. These values were all lower than the similarity of 98.65%, which was proposed by Kim et al. as the threshold for differentiating two species (Kim et al., 2014). In the phylogenetic tree based on the 16S rRNA gene sequences of all genera within the order *Rhodospirillales*, strains CPCC 101082^T^ and CPCC 101083^T^ fell in the genus *Geminicoccus* lineage and formed a robust unique cluster with *Geminicoccus roseus* DSM 18922^T^ in the neighbor-joining tree (Figure 1), which showed almost the same case in the maximum-parsimony tree and maximum-likelihood tree. These results suggested that strains CPCC 101082^T^ and CPCC 101083^T^ represent two novel species of the genus *Geminicoccus*.

### Phenotypic properties

#### Morphological and physiological characteristics

Strains CPCC 101082^T^ and CPCC 101083^T^ grew well on GYM agar. Better growth was observed under light compared with dark conditions. White/light yellow colonies with a wrinkled surface and a maximum diameter of 3–5 mm with irregular edges on GYM were observed after incubation in an illumination incubator for 8 days at 30°C. Strain CPCC 101083^T^ formed pale pink colonies with a slightly wrinkled surface and a maximum diameter of 1–3 mm with regular edges on GYM after incubation in an illumination incubator for 8 days at 30°C. The cells of strains CPCC 101082^T^ and CPCC 101083^T^ were gram-negative, coccoid or short-rods. Both strains were aerobic. The absorption spectra of pigments extracted from the cells of strain CPCC 101083^T^ exhibited the same as of carotenoids, with major absorption maxima at 467, 498 and 525 nm.

The growth of the strain CPCC 101082^T^ occurred at 15–42°C (optimum 28–37°C), and pH 4.0–10.0 (optimum pH 6.0–8.0). The range of NaCl tolerance was 0–4% (w/v). Strain CPCC 101083^T^ grew at 4–45°C (optimum 25–30°C) and pH 4.0–10.0 (optimum pH 6.0–8.0). The range of NaCl for growth was 0–5% (w/v).

In the fermentation broth of strains CPCC 101082^T^ and CPCC 101083^T^, 3-indole-acetic acid (IAA) was detected. As shown in Supplementary Figure S1, the linear regression equation *y* = 0.0244*x* + 0.0482, *r*^2^ = 0.9998, had a good fit. Accordingly, the IAA content produced by the test strains could be calculated according to this equation. The IAA content yielded from the fermentation broth of CPCC 101082^T^, CPCC 101083^T^ and DSM 18922^T^ was 3.83 ± 0.02, 4.75 ± 0.09, and 2.10 ± 0.05 mg/L, respectively.

The activity of alkaline phosphatase of strain CPCC 101082^T^ and CPCC 101082^T^ was positive. The endoglucanase activity of strain CPCC 101082^T^ was positive, while the endoglucanase activity of strain CPCC 101083^T^ was weakly positive. Strain CPCC 101082^T^ could hydrolyze urea, while CPCC 101083^T^ could not. The activities of nitrate reduction, oxidase and catalase of strain CPCC 101083^T^ were positive, but those of strain CPCC 101082^T^ were negative. Other detailed physiological characteristics and the differential phenotypic characteristics of strains CPCC 101082^T^ and CPCC 101083^T^ with respect to the closest phylogenetic neighbor *G. roseus* DSM 18922^T^ and other phylogenetic neighbors are shown in Table 1.

**Table 1:** Differentiating phenotypic characteristics of strain CPCC 101082^T^, CPCC 101083^T^, and *Geminicoccus roseus* DSM 18922^T^.

Characteristic	CPCC 101082^T^	CPCC 101083^T^	DSM 18922^T^
Colony color	white to light yellow	pale pink	whitish-grey to light pink
Cell morphology	coccoid to rod	coccoid to short rod	coccoid
Temperature range for growth (°C; Optimum)	15–42 (28–37)	4–45 (25–30)	15–45 (30–35)
pH range for growth (Optimum)	4.0–10.0 (6.0–8.0)	4.0–10.0 (6.0–8.0)	5.5–11.0 (6.0–7.0)
Tolenrance of NaCl (%)	0–4	0–5	0–10
**Hydrolysis of**			
Urea	+	−	+
Gelatin	−	−	+
Nitrate reduced to nitrite	−	+	+
Oxidase	−	+	−
Catalase	−	+	+
Degradation of cellulose	+	w	−
**Enzyme activities (API ZYM)**			
Leucine arylamidase	+	+	w
Valine arylamidase	+	+	−
Cystine arylamidase	+	+	−
Trypsin	+	w	−
*α*-chymotrypsin	w	−	−
Acid phosphatase	−	w	+
*β*-glucuronidase	−	+	−
**Utilization of (Biolog GEN III):**			
Dextrin	w	w	−
D-Maltose	w	−	−
D-Cellobiose	+	w	−
Gentiobiose	+	−	−
*α*-D-Lactose	w	−	−
D-Melibiose	w	−	−
N-Acetyl-D-Glucosamine	−	−	+
*α*-D-Glucose	+	+	−
D-Mannose	+	+	−
D-Galactose	+	+	−
3-Methyl-Glucose	+	+	−
L-Rhamnose	+	+	−
D-Sorbitol	−	+	−
D-Mannitol	−	+	−
D-Arabitol	−	+	−
myo-Inositol	−	+	+
L-Histidine	−	+	−
D-Gluconic Acid	−	+	+
D-Glucuronic Acid	+	−	−
L-Lactic Acid	−	+	+
*α*-Keto-Glutaric Acid	−	−	+
D-Malic Acid	−	−	+
L-Malic Acid	−	−	+
Tween 40	w	−	−
*β*-Hydroxy-D,L-butyric Acid	−	+	+
Acetoacetic Acid	−	+	+
Formic Acid	+	+	−
**Fatty acids profiles (>5%)**	cyclo-C_19:0_ *ω*8*c* (40.4%); C_18:1_ *ω*7*c* and/or C_18:1_ *ω*6*c* (29.0%); C_16:0_ (10.8%)	C_18:1_ *ω*7*c* and/or C_18:1_ *ω*6*c* (37.4%); cyclo-C_19:0_ *ω*8*c* (31.8%); C_16:0_ (14.1%); C_18:0_ (8.9%)	C_18:1_ *ω*7*c* and/or C_18:1_ *ω*6*c* (50.6%); cyclo-C_19:0_ *ω*8*c* (19.1%); C_16:0_ (14.0%)

All strains are Gram-reaction-negative, positive for hydrolysis of starch and production of indole-3-acetic acid, negative for production of indole and H_2_S, VP and MR tests. In API ZYM kits, all strains are positive for alkaline phosphatase, esterase (C_4_), esterase lipase (C_8_), leucine arylamidase and naphthol-AS-B1-phosphohydrolase, negative for lipase (C_14_), α-galactosidase, β-galactosidase, α-glucosidase, β-glucosidase, N-acetyl-β-glucosaminidase, α-mannosidase and β-fucosidase. In Biolog GEN III (MicroPlate), all strains are positive for D-fructose, D-fucose, L-fucose, D-glucose-6-PO_4_, D-fructose-6-PO_4_, D-galacturonic acid, L-galactonic acid lactone, glucuronamide and acetic acid, are negative for D-trehalose, sucrose, D-turanose, stachyose, D-raffinose, β-methyl-D-glucoside, D-salicin, N-acetyl-β-D-mannosamine, N-acetyl-D-galactosamine, N-acetyl-neuraminic acid, inosine, glycerol, D-aspartic acid, D-serine, gelatin, glycyl-L-proline, L-alanine, L-arginine, L-aspartic acid, L-glutamic acid, L-pyroglutamic acid, L-serine, pectin, mucic acid, quinic qcid, D-saccharic acid, p-hydroxy-phenylacetic acid, methyl pyruvate, D-lactic acid methyl ester, citric acid, bromo-succinic acid, γ-amino-butryric acid, α-hydroxy-butyric acid, α-keto-butyric acid and propionic acid. All data were from this study. +, positive; −, negative; w, weakly positive.

#### Chemotaxonomic characteristics

The polar lipids of strains CPCC 101082^T^ and CPCC 101083^T^ mainly consisted of diphosphatidylglycerol (DPG), phosphatidylglycerol (PG), phosphatidylcholine (PC), phosphatidylethanolamine (PE), an unidentified phospholipid (PL) and an unidentified aminolipid (AL; Supplementary Figure S2). The sole respiratory quinone was detected as Q-10. The fatty acids (>5%) of strain CPCC 101082^T^ contained cyclo-C_19:0_*ω*8*c* (40.4%), Summed Feature 8 (C_18:1_*ω*7*c*/C_18:1_*ω*6*c*; 29.0%) and C_16:0_ (10.8%) with minor (1–5%) amounts of C_17:1_*ω*6*c* (4.5%), Sum In Feature 4 (iso-C_17:1_ I and/or anteiso-C_17:1_ B; 2.2%), Sum In Feature 3 (C_16:1_ ω7c and/or iso-C_15:0_ 2-OH; 2.1%), C_18:1_*ω*5*c* (2.0%), C_18:0_ (1.8%) and C_16:0_3-OH (1.8%). The major fatty acids (>5%) of strain CPCC 101083^T^ were Summed Feature 8 (C_18:1_*ω*7*c*/C_18:1_*ω*6*c*; 37.4%), cyclo-C_19:0_*ω*8*c* (31.8%), C_16:0_ (14.1%) and C_18:0_ (8.9%), with minor (1–5%) amounts of C_18:0_ 3-OH (1.8%; Supplementary Table S1).

The profile of the chemotaxonomic properties stated above well supported the classification of strains CPCC 101082^T^ and CPCC 101083^T^ as two members of the genus *Geminicoccus*. However, strains CPCC 101082^T^ and CPCC 101083^T^ differed from each other and as well with the only validly identified *Geminicoccus* member *G. roseus* DSM 18922^T^ in some physiological properties and the cellular fatty acid profile (Table 1; Supplementary Table S1).

Filled circles indicate that the corresponding nodes were also recovered in the trees generated with the maximum-likelihood and maximum-parsimony methods. Bootstrap values (>50%) are shown as percentages of 1,000 replicates. Bar, 2 nt substitutions per 100 nt.

### Genome properties

Genome sequencing of strain CPCC 101082^T^ yielded a draft genome containing 5,871,762 bp, assembled from 1,148 qualified scaffolds, with a coverage of 309-fold and an N50 length of 8,840 bp. Strain CPCC 101083^T^ had the following characteristics: a draft genome of 5,893,129 bp, assembled from 286 qualified scaffolds, with a coverage of 384-fold and an N50 length of 180,117 bp. The G + C content in the genomic DNA of strains CPCC 101082^T^ and CPCC 101083^T^ was calculated as 68.2 and 67.9%, respectively, from the genome sequence. The dDDH values among strains CPCC 101082^T^, CPCC 101083^T^, and *Geminicoccus roseus* DSM 18922^T^ ranged from 23.0 to 47.0%, far below the cut-off value (70%) used to classify bacterial strains of the same species (Auch et al., 2010); the ANI values among CPCC 101082^T^, CPCC 101083^T^ and *Geminicoccus roseus* DSM 18922^T^ ranged from 80.6 to 92.4%, also much lower than the threshold for bacterial species delineation (95–96%; Kim et al., 2014; Supplementary Table S2).

**Figure 2:**
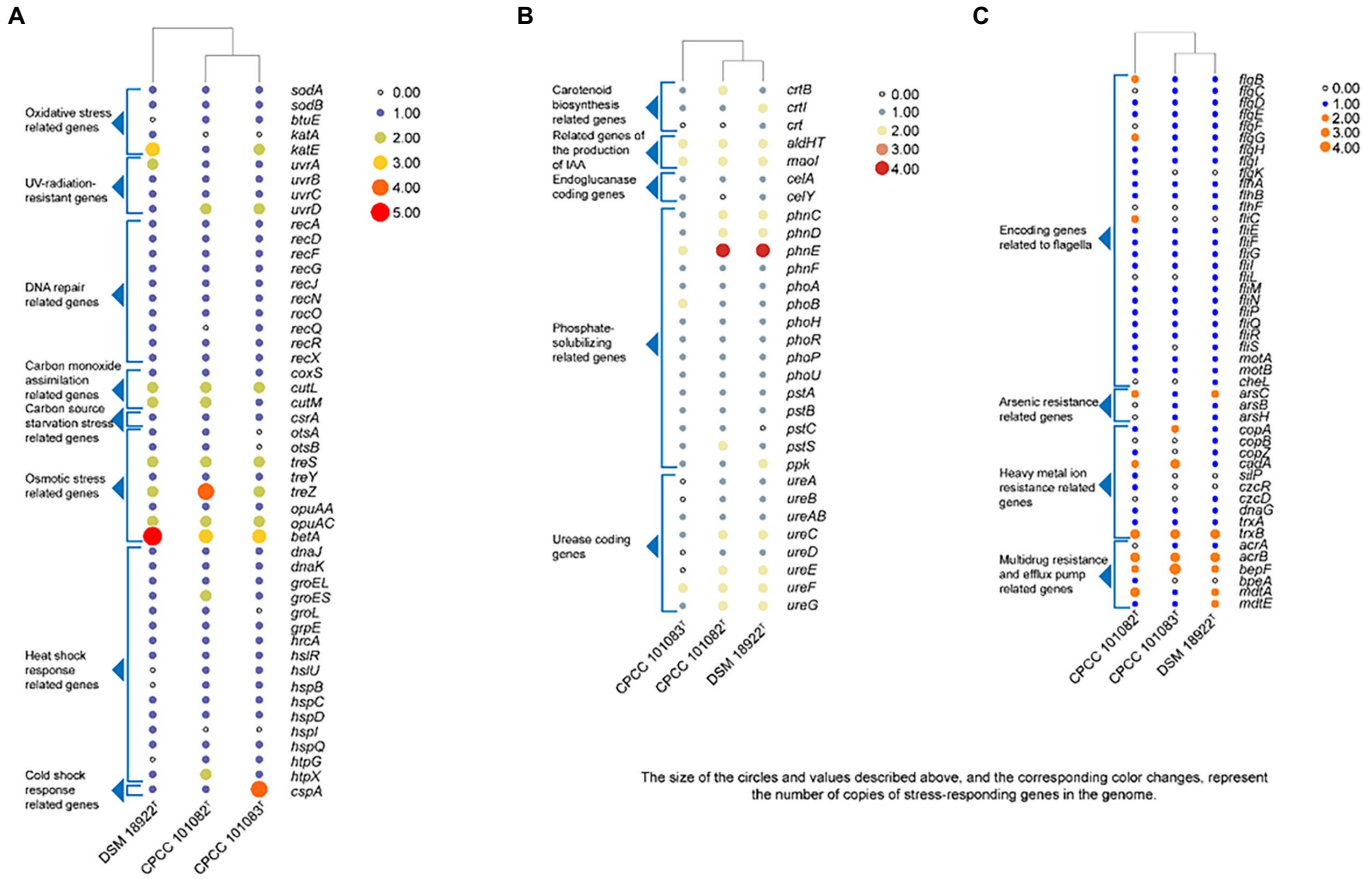
Heatmap of putative functional genes predicted in the genomes of strains CPCC 101082^T^, CPCC 101083^T^ and their closest phylogenic neighbor *Geminicoccus roseu* DSM 18922^T^ according to the copy number of the genes from the KEGG annotation. **(A)** Stress-responding related genes. **(B)** Metabolism related genes. **(C)** Encoding genes related to flagella, arsenic resistance, heavy metal ion resistance, multidrug resistance and efflux pump.

The whole genome of strains CPCC 101082^T^ and CPCC 101083^T^ contained 5,733 and 5,396 genes, respectively (Table 2). The strain *G. roseus* DSM 18922^T^, which was reported to be isolated from the biofilter of a recirculating marine aquaculture system composed of 5,260 genes, was included as a reference. The detailed genomic characteristics of these strains are summarized in Table 2. In the genomes of strains CPCC 101082^T^, CPCC 101083^T^ and *G. roseus* DSM 18922^T^, the putatively encoding genes for superoxide dismutase (*sodA* and *sodB*), catalase (*kata* and *katE*), peroxidase (*btuE*) and UV radiation-resistant genes (*uvrA*, *uvrB*, *uvrC*, and *uvrD*) were retrieved, which might endow these strains the ability to adapt to the strong solar radiation in the desert. The DNA repair-related genes (*recA*, *recD*, *recF*, *recG*, *recJ*, *recN*, *recO*, *recQ*, *recR*, and *recX*), heat shock-related genes (*dnaJ*, *dnaK*, *groEL*, *groES*, *groL*, *grpE*, *hrcA*, *hslR*, *hslU*, *hspB*, *hspC*, *hspD*, *hspI*, *hspQ*, *htpG*, and *htpX*), cold shock-related genes (*cspA*), carbon monoxide assimilation-related genes (*coxS*, *cutL* and *cutM*), carbon source starvation response-related genes (*csrA*) and osmotic stress-related genes (*otsA*, *otsB*, *treS*, *treY*, *treZ*, *opuAA*, *opuAC*, and *betA*) were also retrieved. These genes mentioned above were related to the microorganisms response to the abiotic-stress in extreme desert environment. The metabolism related genes were retrieved, such as carotenoid biosynthesis-related genes (*crtB*, *crtI*, and *crt*), endoglucanase-coding genes (*celA* and *celY*), urease coding genes (*ureA*, *ureB*, *ureAB*, *ureC*, *ureD*, *ureE*, *ureF*, and *ureG*), genes related to the production of indole-3-acetic acid (*aldHT*, *maoI*) and phosphate-solubilizing genes (*phnC*, *phnD*, *phnE*, *phnF*, *phoA*, *phoB*, *phoH*, *phoR*, *phoP*, *phoU*, *pstA*, *pstB*, *pstC*, *pstS*, and *ppk*). In addition, genes encoding flagella (*flgB*, *flgC*, *flgD*, *flgE*, *flgF*, *flgG*, *flgH*, *flgI*, *flgK*, *flhA*, *flhB*, *flhF*, *fliC*, *fliE*, *fliF*, *fliG*, *fliI*, *fliL*, *fliM*, *fliN*, *fliP*, *fliQ*, *fliR*, *fliS*, *motA*, *motB*, and *cheL*), arsenic resistance-related genes (*arsB*, *arsC*, and *arsH*), heavy metal ion resistance-related genes (*copA*, *copB*, *copZ*, *cadA*, *silP*, *czcR*, *czcD*, *dnaG*, *trxA*, and *trxB*) and multidrug resistance and efflux pump-related genes (*acrA*, *acrB*, *bepF*, *bpeA*, *mdtA*, *mdtE*, and *mexG*) were also retrieved from the genomes of strains CPCC 101082^T^, CPCC 101083^T^ and *G. roseus* DSM 18922^T^ (Figure 2).

**Table 2:** Basic characteristics of the genomes of strains CPCC 101082^T^, CPCC 101083^T^ and *Geminicoccus roseus* DSM 18922^T^.

Functional genes	CPCC 101082^T^	CPCC 101083^T^	DSM 18922^T^
**The genomic characteristics**			
Genome size (Mbp)	5.9	5.9	5.7
Scaffold (s)	1,148	286	5
N50 Length (bp)	8,840	180,117	5,421,495
Genome coverage (%)	99.4	99.9	100
G + C (%)	68.2	67.9	68.5
16S rRNA gene length (bp) retrieved from the assembly genome	1,449	1,453	1,407
Genes (CDSs)	5,733	5,396	5,260
DDBJ/EMBL/GenBank accession number of darft genome	WUJP00000000	JABGCL000000000	ATYL00000000
GenBank/RefSeq assembly accession number	GCA_009806515.1	GCA_019800005.1	GCA_000427665.1

The assembly sequences of the strains CPCC 101082^T^ and CPCC 101083^T^ were derived from this study. The assembly sequence of the strain *Geminicoccus roseus* DSM 18922^T^ was downloaded from NCBI.

### Pan-genome analysis of the genus *Geminicoccus*

**Figure 3:**
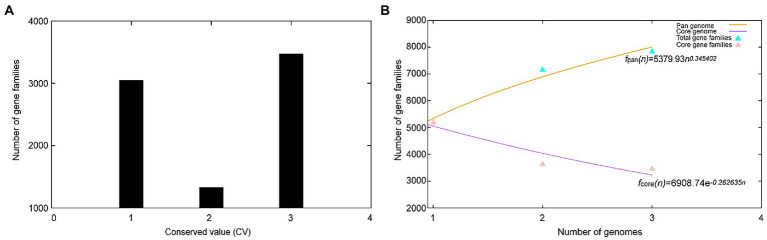
Overview of the pan-genomic results generated by BPGA using 3 strains of the genus *Geminicoccus*. **(A)** The gene family frequency spectrum. **(B)** The pan genome profile trends of the genus *Geminicoccus* obtained using clustering tools USEARCH.

A total of 17,468 protein-coding genes (Table 3) were sorted from the genomes of these three strains of the genus *Geminicoccus*, which were divided into 7,856 homologous families by cluster analysis. Histograms were constructed according to different CVs (Figure 3A). Among them, there were a total of 3,472 core genes commonly shared by these three strains (CV = 3), accounting for about 44.2% of the total number of homologous gene families. The accessory genes (1,334 genes; CV = 2) accounted for about 17.0% of the homologous gene families in the newly proposed species. The proportion of the unique genes (3,050 genes; CV = 1) was about 38.8%.

**Table 3:** The pan-genome profile information of the genus *Geminicoccus.*

Genome number	Organism name	No. of core genes	No. of accessory genes	No. of unique genes	No. of exclusively absent genes
1	DSM 18922^T^	3,472	316	1,421	1,018
2	CPCC 101082^T^	3,472	1,187	941	147
3	CPCC 101083^T^	3,472	1,165	688	169

The relationship between the pan-genome size and the number of genomes of the genus and the relationship between the number of core genes and the number of genomes were deduced (Figure 3B) using all of the protein sequences extracted from these three strains of the genus *Geminicoccus*. The functional relationship between the pan-genome size (f_pan_) and the number of genomes (n) was obtained by fitting, as follows:


$$f_{\mathrm{pan}}\;\left(n\right)=5379.93\times n^{0.345402}$$

The functional relationship between the number of core genes (f_core_) and the number of genomes (n) was obtained by fitting, as follows:


$$f_{\mathrm{core}}\;\left(n\right)=6908.74\times e^{-0.262635n}$$

It could be inferred from the pan-genome fitting curve in Figure 3B that, with the increasing number of sequenced genomes, the pan-genome size became larger and larger, instead of tending to a plateau. Accordingly, it could be proposed that the pan-genome of the genus *Geminicoccus* is the open type, which suggests that the genus *Geminicoccus* has a strong ability to accept horizontal gene transfers.

**Figure 4:**
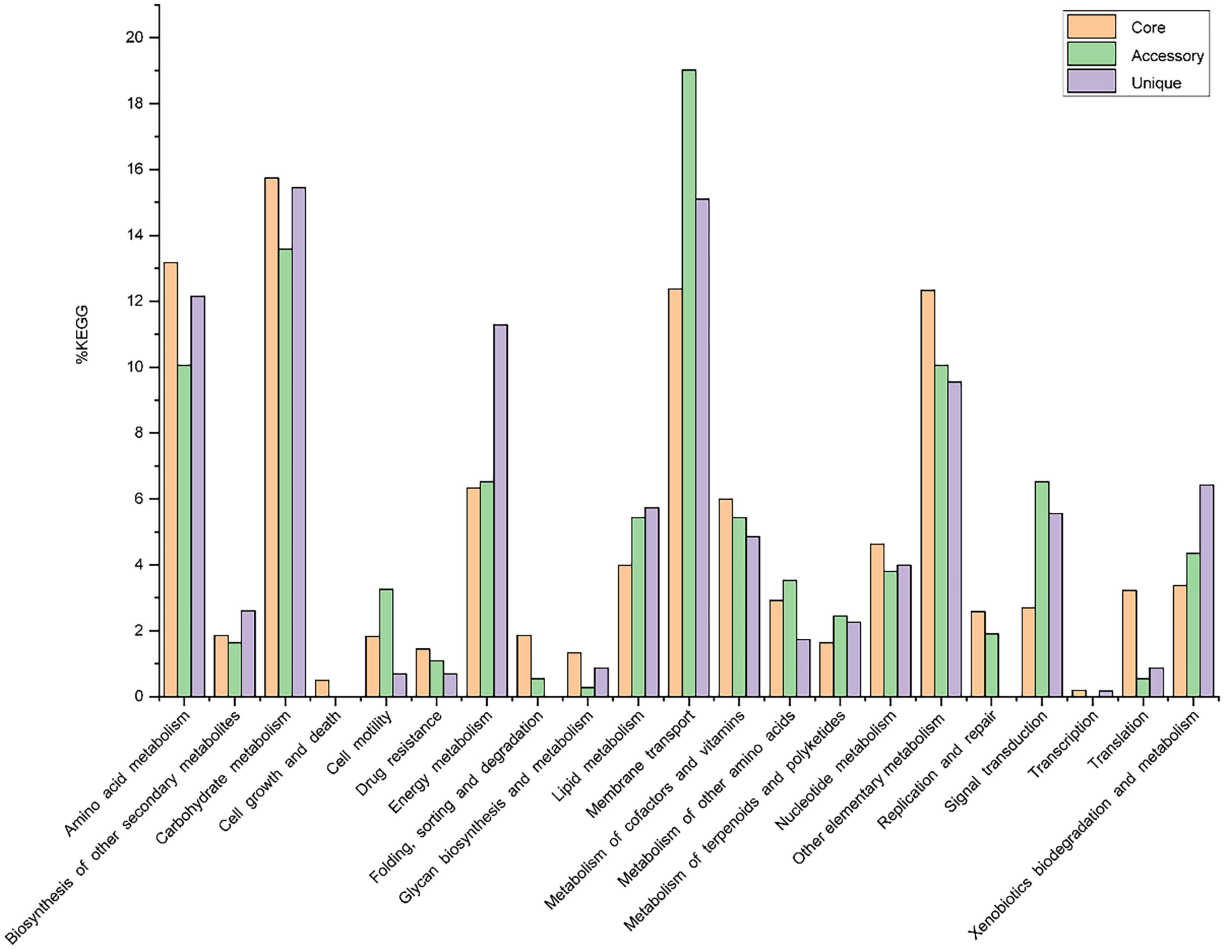
The assigned metabolic pathways associated with the core, accessory and unique genes among the genus *Geminicoccus* from the KEGG database.

Out of 7,856 genes/gene clusters, BPGA mapped 3,911 (49.8%) to KEGG (Kyoto Encyclopedia of Genes and Genomes) pathways, i.e., core gene (2,860, 73.1%), accessory genes (437, 11.2%) and unique genes (614, 15.7%). After filtering KEGG pathways related to eukaryotes, we obtained an overview of the metabolic pathway corresponding to the gene(s) in the pan-genome of the genus *Geminicoccus.* A large number of core genes (2,635) were involved in carbohydrate metabolism (15.8%), amino acid metabolism (13.2%), membrane transport (12.4%), some other elementary metabolism (biosynthesis of amino acids, 5.1%; carbon metabolism, 4.0%; fatty acid metabolism, 1.4%; 2-oxocarboxylic acid metabolism, 1.4% and degradation of aromatic compounds, 0.4%; 12.3%), energy metabolism (6.3%), metabolism of cofactors and vitamins (6%), nucleotide metabolism (4.6%), lipid metabolism (4.0%), xenobiotic biodegradation and metabolism (3.4%), translation (3.2%), metabolism of other amino acids (2.9%), signal transduction (2.7%), replication and repair (2.6%), biosynthesis of other secondary metabolites (1.9%), folding, sorting and degradation (1.9%), cell motility (1.8%), metabolism of terpenoids and polyketides (1.6%), drug resistance (1.4%), glycan biosynthesis and metabolism (1.3%). Accessory and unique genes appeared to be enriched in membrane transport, carbohydrate metabolism, amino acid metabolism, as well as energy metabolism. Among the accessory genes (368), the major portion of genes were related to membrane transport (19.0%), carbohydrate metabolism (13.6%), some other elementary metabolism (biosynthesis of amino acids, 3.0%; carbon metabolism, 2.8%; fatty acid metabolism, 2.4%; degradation of aromatic compounds, 1.1% and 2-oxocarboxylic acid metabolism, 0.8%; 10.1%), amino acid metabolism (10.1%), signal transduction (6.5%), energy metabolism (6.5%), lipid metabolism (5.4%), metabolism of cofactors and vitamins (5.4%), xenobiotics biodegradation and metabolism (4.4%), nucleotide metabolism (3.8%), metabolism of other amino acids (3.5%), cell motility (3.3%), metabolism of terpenoids and polyketides (2.5%), replication and repair (1.9%), biosynthesis of other secondary metabolites (1.6%) and drug resistance (1.1%). Unique genes (576) seemed to be mainly enriched in carbohydrate metabolism (15.5%), membrane transport (15.1%), amino acid metabolism (12.2%), energy metabolism (11.3%), xenobiotics biodegradation and metabolism (6.4%), lipid metabolism (5.7%), signal transduction (5.6%), metabolism of cofactors and vitamins (4.9%), nucleotide metabolism (4.0%), biosynthesis of other secondary metabolites (2.6%), metabolism of terpenoids and polyketides (2.3%) and the metabolism of other amino acids (1.7%; Figure 4).

In the phylogenetic trees based on the concatenated core genes and binary gene presence/absence matrix (pan-matrix), strains CPCC 101082^T^ and CPCC 101083^T^ clustered in a unique branch in the lineage of the genus *Geminicoccus*, with the closest evolutionary distance with the branch formed by *G. roseus* DSM 18922^T^ (Supplementary Figures S3, S4).

### Speculation of metabolic characteristics

Two KEGG pathways for autotrophic carbon dioxide fixation of *strains* CPCC 101082^T^ and CPCC 101083^T^ were predicted (Supplementary Figure S5). The KEGG pathway diagram showed that the genes of strains CPCC 101082^T^ and CPCC 101083^T^ encoded some enzymes in autotrophic carbon dioxide fixation pathways in prokaryotes other than in the reductive pentose phosphate pathway (Supplementary Figures S5A,C). The KEGG pathway diagram also showed that the genes of strains CPCC 101082^T^ and CPCC 101083^T^ encoded some enzymes in the reductive pentose phosphate pathway of autotrophic carbon dioxide fixation (Supplementary Figures S5B,D). In the predicted KEGG pathway of the strain CPCC 101082^T^ and CPCC 101083^T^, some multidrug resistance and efflux pump related coding genes were retrieved, for example, *acrA* and *acrB* (multidrug efflux pump subunit AcrA and AcrB coding gene; Wang et al., 2017), *bepF* (Martin et al., 2009; efflux pump periplasmic linker BepF coding gene, may contribute to resistance to some drugs, such as sodium dodecyl sulfate and nalidixic acid), *bpeA* (multidrug efflux periplasmic linker protein BpeA coding gene), *mdtA* (Nagakubo et al., 2002) and *mdtE* (multidrug resistance protein MdtA and MdtE coding gene) and *mexG* (Aires et al., 1999; multidrug efflux RND transporter inhibitory subunit MexG coding gene).

### Taxonomic study conclusion

The phenotypic and genotypic data supported the accommodation of strains CPCC 101082^T^ and CPCC 101083^T^ in the genus *Geminicoccus* and also distinguished these two strains from each other as well from other species of the genus *Geminicoccus.* Accordingly, we propose that strains CPCC 101082^T^ and CPCC 101083^T^ be classified as representatives of two new species of the genus *Geminicoccus*, with the names *Geminicoccus flavidas* sp. nov. and *Geminicoccus harenae* sp. nov.

### Description of *Geminicoccus flavidas* sp. nov.

*Geminicoccus flavidas* (fla′vi.da. L. fem. adj. *flavida* yellowish).

Cells are Gram-staining-negative, coccoid to rods, non-motile and aerobic. Grows well on GYM agar and nutrient agar. Colonies on GYM agar are white/light yellow colonies with wrinkled, circular, convex, and opaque, approximately 1 mm in diameter after 5 days at 30°C (pH 7.0). Grows at 15–42°C and pH 4.0–10.0, with the optimum at 28–37°C and pH 6.0–8.0, respectively. Grows in NaCl at concentrations up to not more than 4%. Can utilize 3-methyl glucose, acetic acid, D-cellobiose, dextrin, D-fructose, D-fructose-6-PO_4_, D-fucose, D-galactose, D-galacturonic acid, D-glucose-6-PO_4_, D-glucuronic acid, D-maltose, D-mannose, D-melibiose, formic acid, gentiobiose, glucuronamide, L-fucose, L-galactonic acid lactone, L-rhamnose, tween 40, *α*-D-glucose and *α*-D-lactose as the sole carbon source, and D-arabinose, D-arabitol, D-ardonitol, D-fructose, D-fucose, D-galactose, D-glucose, D-lyxose, D-mannitol, D-mannose, D-ribose, D-sorbitol, D-tagatose, dulcitol, D-xylose, esculin ferric citrate, inositol, L-arabinose, L-arabitol, L-fucose, L-rhamnose, L-sorbose, L-xylose, potassium 5-keto-gluconate and xylitol can be assimilated to produce acid. Positive for alkaline phosphatase, cystine arylamidase, esterase (C4), esterase lipase (C8), leucine arylamidase, naphthol-AS-BI-phosphohydrolase, trypsin and valine arylamidase, weakly positive for *α*-chymotrypsin in API ZYM strip. The cellular polar lipid system includes diphosphatidylglycerol (DPG), phosphatidylglycerol (PG), phosphatidylcholine (PC), phosphatidylethanolamine (PE), an unidentified phospholipid (PL) and an unidentified aminolipid (AL). The sole respiratory quinone is Q-10. The major cellular fatty acids include cyclo-C_19:0_*ω*8*c*, C_18:1_*ω*7*c*/C_18:1_*ω*6*c*, and C_16:0_. The genome of the type strain is characterized by a size of 5.95 Mbp and the G + C content of 68.2%. The type strain CPCC 101082^T^ (=NBRC 113513^T^ =KCTC 62853^T^) was isolated from a hinterland area characterized by cyanobacteria-dominated crusts collected from Badain Jaran desert, China. The DDBJ/EMBL/GenBank accession numbers of 16S rRNA gene sequence and draft genome sequence of the type strain are MK392026 and WUJP00000000, respectively.

### Description of *Geminicoccus harenae* sp. nov.

*Geminicoccus harenae* (ha.re′nae. L. gen. n. *harenae* of sand, of a desert, referring to the isolation source of the type strain from desert sand).

Cells are Gram-reaction-negative, coccoid to short-rods, non-motile and aerobic. Grows well on GYM agar and nutrient agar. Colonies on GYM agar are wrinkled, circular, convex, and opaque and opaque with a pale pink color, approximately 1 mm in diameter after 5 days at 30°C (pH 7.0). Grows at 4–45°C and pH 4.0–10.0, with the optimum at 25–30°C and pH 6.0–8.0. NaCl is not necessary for growth, while NaCl tolerance is 5.0% (w/v). Can utilize 3-methyl glucose, acetic acid, acetoacetic acid, D-arabitol, D-cellobiose, dextrin, D-fructose, D-fructose-6-PO_4_, D-fucose, D-galactose, D-galacturonic acid, D-gluconic acid, D-glucose-6-PO_4_, D-mannitol, D-mannose, D-sorbitol, formic acid, glucuronamide, L-fucose, L-galactonic acid lactone, L-histidine, L-lactic acid, L-rhamnose, myo-inositol, *α*-D-glucose and *β*-hydroxy-D,L-butyric acid as the sole carbon source, and amygdalin, arbutin, D-arabinose, D-arabitol, D-ardonitol, D-cellobiose, D-fructose, D-fucose, D-galactose, D-glucose, D-lyxose, D-mannitol, D-mannose, D-ribose, D-sorbitol, D-tagatose, D-trehalose, dulcitol, D-xylose, esculin ferric citrate, inositol, L-arabinose, L-fucose, L-rhamnose, L-sorbose, L-xylose, N-acetyl-glucosamine, potassium 2-ketogluconate, potassium 5-ketogluconate, salicin and xylitol can be assimilated to produce acid. Positive alkaline phosphatase, cystine arylamidase, esterase(C4), esterase lipase(C8), leucine arylamidase, naphthol-AS-B1-phosphohydrolase, valine arylamidase and *β*-glucuronidase, weakly positive for acid phosphatase and trypsin in API ZYM strip. The predominant polar lipids are diphosphatidylglycerol (DPG), phosphatidylglycerol (PG), phosphatidylcholine (PC), phosphatidylethanolamine (PE), an unidentified phospholipid (PL) and an unidentified aminolipid (AL). The sole respiratory quinone is Q-10. The major cellular fatty acids are C_18:1_*ω*7*c*/C_18:1_*ω*6*c*, cyclo-C_19:0_*ω*8*c*, C_16:0_ and C_18:0_. The genome sequence is characterized by a size of 6.02 Mbp and the G + C content of 67.3%. The type strain CPCC 101083^T^ (=NBRC 113514^T^ = KCTC 62853^T^) was isolated from a sandy dune sample with moss-dominated crusts collected from Badain Jaran desert, China. The DDBJ/EMBL/GenBank accession numbers of 16S rRNA gene sequence and draft genome sequence of the strain CPCC 101083^T^ are MK392027 and JABGCL000000000, respectively.

## Discussion

Based on the genomic information, we summarized the genetic characteristics of the novel species *G. flavidas* sp. nov. and *G. harenae* sp. nov. accommodating the type strain recovered from a desert. The detailed phenotypic properties illustrated the abilities of these strains to adapt to harsh environmental stress. The genome-scale analysis of strains CPCC 101082^T^ and CPCC 101083^T^ indicated that adaptation of these organisms to desert niches was achieved through various response strategies to UV radiation, carbon starvation, desiccation, osmotic stress and so on.

Dehydration of bacterial cells due to desiccation caused severe damage to enzymes and electron transport chains, leading to the accumulation of free radicals (Billi and Potts, 2002), which in turn seriously damaged DNA (Dose et al., 1992). It is proposed that extreme radiation resistance in desert microorganisms is functionally related to the photochemical oxidation of desiccated cells. In such environments, aerobic organisms have antioxidant enzymes, such as superoxide dismutase, catalase and peroxidase, and some UV-radiation resistant related genes that are helpful in protecting metabolically active cells against endogenous reactive oxygen species generated from aerobic respiration. In the core genome of the genus *Geminicoccus*, 68 gene clusters (2.6%) were mapped to replication and repair (KO09124, a KEGG sub-category), consisting of DNA replication (ko03030), base excision repair (ko03410), nucleotide excision repair (ko03420), mismatch repair (ko03430), and homologous recombination (ko03440) et al. In the annotation results, some oxidative stress related genes, such as superoxide dismutase coding genes (*sodA* and *sodB*), thioredoxin/glutathione peroxidase (*btuE*), catalase coding genes (*katA* and *katE*), some UV-radiation resistant genes, such as the UvrABC repair system coding genes (*uvrA, uvrB,* and *uvrC*), DNA helicase II coding gene (*uvrD*) and some DNA repair related genes (*recA*, *recD*, *recF*, *recG*, *recJ*, *recN*, *recO*, *recQ*, *recR*, and *recX*) were retrieved (Figure 2).

**Figure 5:**
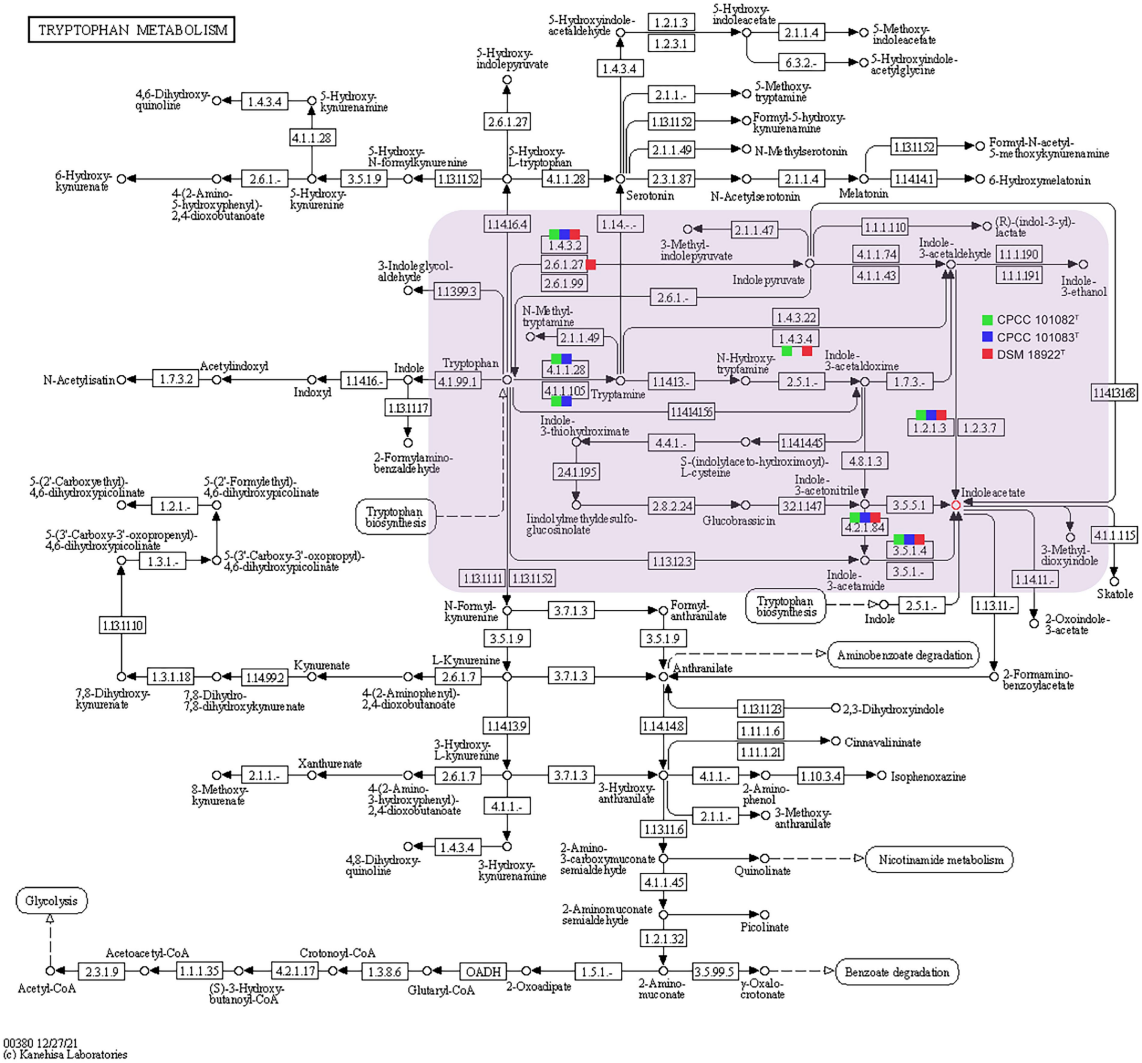
Putative overview of indole-3-acetic acid-producing pathway in the tryptophan metabolism pathways of the strain CPCC 101082^T^, CPCC 101083^T^, and *Geminicoccus roseu* DSM 18922^T^. Squares colored with green, blue and red represent the strain CPCC 101082^T^, CPCC 101083^T^, and DSM 18922^T^, respectively.

In addition to mediating plant-microbe interaction, recent studies have shown that IAA conferred protection against environmental stresses such as UV radiation, salt, acidity, osmotic shock and heat shock (Bianco et al., 2009; Duca et al., 2014). The increasing trehalose, LPS (lipopolysaccharide), EPS (exopolysaccharide) and biofilm content induced by IAA enhanced the resistance of bacteria to environmental stress (Bianco et al., 2009). Therefore, these novel and rare IAA-producing bacterial species in desert habitats, may promote the colonization of other dominant bacteria in desert habitats. The phenotypic assays confirmed that all the strains CPCC 101082^T^, CPCC 101083^T^ and DSM 18922^T^ could produce IAA. In the genomic category, the aldehyde dehydrogenase (EC 1.2.1.3) coding genes and amine oxidase coding gene (*maoI*) were retrieved from the genomes of CPCC 101082^T^, CPCC 101083^T^ and *G. roseu* DSM 18922^T^. In addition, according to the distribution of IAA-producing genes among the three strains, the pathway of IAA production (Figure 5) was predicted through the annotation of the KEGG database.

The strain CPCC 101082^T^ could hydrolyze urea, but CPCC 101083^T^ could not. The difference in the copy numbers of urease-encoding genes might be the key factor leading to the difference in urea hydrolysis activity between the two strains. The carotenoid biosynthesis-related gene (*crtB*) was retrieved from the genome of strain CPCC 101083^T^, not CPCC 101082^T^, which could help us understand that the methanol/acetone extracts from CPCC 101083^T^ displaying absorbance maxima (418, 480 and 537 nm) were indicative of carotenoids other than those from strain CPCC 101082^T^. Bacterial secondary metabolism is a rich source of novel bioactive compounds with potential medicinal value. To find new drug-producing candidates, microbiologists are increasingly employing genome sequencing of a wide variety of microbes. Here, we identified biosynthetic sites by antiSMASH (antibiotic &Secondary Metabolite Analysis Shell.^10^ However, the results from the antiSMASH database showed that in the two strains, *G. flavidas* sp. nov. and *G. harenae* sp. nov., only 10 secondary metabolite gene clusters with moderate similarities to previously described secondary metabolite biosynthetic gene clusters were retrieved (Supplementary Table S3). These gene clusters exhibited 5–32% similarities to previously reported secondary metabolite biosynthetic gene clusters, such as bacillibactin NRP, ibomycin (Polyketide), NRP: cyclic depsipeptide + polyketide: Modular type I, oxalomycin B, alkaloid gene clusters and other unidentified secondary metabolite clusters attributable to NAPAA, terpene, thioamitides, RRE-containing and T1PKS types. Abundant functional genes (for instance, stress-responding genes) were found in the core genomes of the newly proposed species, *G. flavidas* and *G. harenae*, which are assumed to contribute greatly to their survival abilities in harsh desert environments. Therefore, from an evolutionary perspective, the ability to endure harsh environments might endow the bacteria with advantages in coping with desert environments; being the indispensable constituents of the pioneer biological population in the desert environment, these microorganisms contributed greatly to the development of the biosphere.

## Data availability statement

The datasets presented in this study can be found in online repositories. The names of the repository/repositories and accession number(s) can be found in the article/Supplementary material.

## Author contributions

Z-MJ, YD, and X-FH carried out the experiments and prepared the manuscript. JS, HW, and L-YY collected the samples. Z-MJ and Y-QZ designed the research and analyzed the data. All authors contributed to the article and approved the submitted version.

## Funding

This research was supported by CAMS Innovation Fund for Medical Sciences (CIFMS, 2021-I2M-1-055), National Natural Science Foundation of China (32170021), Beijing Natural Science Foundation (5212018), Key project at central government level-the ability establishment of sustainable use for valuable Chinese medicine resources (2060302), and the National Infrastructure of Microbial Resources (NIMR-2021-3).

## Conflict of interest

The authors declare that the research was conducted in the absence of any commercial or financial relationships that could be construed as a potential conflict of interest.

## Publisher’s note

All claims expressed in this article are solely those of the authors and do not necessarily represent those of their affiliated organizations, or those of the publisher, the editors and the reviewers. Any product that may be evaluated in this article, or claim that may be made by its manufacturer, is not guaranteed or endorsed by the publisher.

## Footnotes

^1^ https://lpsn.dsmz.de/family/geminicoccaceae

^2^ http://www.ezbiocloud.net/

^3^ http://www.cbcb.umd.edu/software/glimmer/

^4^ http://tandem.bu.edu/trf/trf.html

^5^ http://www5.esu.edu/cpsc/bioinfo/software/GIST/

^6^ http://phast.wishartlab.com/

^7^ https://www.uniprot.org/

^8^ https://www.ebi.ac.uk/interpro/

^9^ https://www.ezbiocloud.net/tools/ani

^10^ https://ggdc.dsmz.de/

^10^ http://antismash.secondarymetabolites.org

## Abbreviations

DPG: Diphosphatidylglycerol
PG: Phosphatidylglycerol
PE: Phosphatidylethanolamine
PC: Phosphatidylcholine
PL: Unidentified phospholipid
AL: Unidentified aminophospholipid
ANI: Average nucleotide identity
dDDH: Digital DNA–DNA hybridization
IAA: Indole-3-acetic acid.
