# Corrigendum: *Geminicoccus flavidas* sp. nov. and *Geminicoccus harenae* sp. nov., two IAA-producing novel rare bacterial species inhabiting desert biological soil crusts

**DOI:** 10.3389/fmicb.2023.1285950

**Published:** 2023-09-27

**Authors:** Zhu-Ming Jiang, Yang Deng, Xue-Fei Han, Jing Su, Hao Wang, Li-Yan Yu, Yu-Qin Zhang

**Affiliations:** ^1^Institute of Medicinal Biotechnology, Chinese Academy of Medical Sciences, Beijing, China; ^2^State Key Laboratory of Dao-di Herb, Beijing, China

**Keywords:** *Geminicoccus flavidas*, *Geminicoccus harenae*, average nucleotide identity, pan-genome, biological soil crusts

In the published article, there was an error regarding the Acknowledgments statement. Prof. Aharon Oren and prof. Bernhard Schink did not propose the species' names in this study. Thus, the statement printed was inappropriate. Consequently, the authors express their sincere apologies to both Prof. Oren and Prof. Schink.

In the published article, there was an error in protologue of the *Geminicoccus harenae* sp. nov.. The KCTC number in the protologue of *Geminicoccus harenae* was incorrectly written as KCTC 62853.

This should have been written as:

*Geminicoccus harenae* (ha.re′nae. L. gen. n. *harenae* of sand, of a desert, referring to the isolation source of the type strain from desert sand).

Cells are Gram-reaction-negative, coccoid to short-rods, non-motile and aerobic. Grows well on GYM agar and nutrient agar. Colonies on GYM agar are wrinkled, circular, convex, and opaque and opaque with a weak pink color, approximately 1 mm in diameter after 5 days at 30°C (pH 7.0). Grows at 4–45°C and pH 4.0–10.0, with the optimum at 25–30°C and pH 6.0–8.0. NaCl is not necessary for growth, while NaCl tolerance is 5.0% (w/v). Can utilize 3-methyl glucose, acetic acid, acetoacetic acid, D-arabitol, D-cellobiose, dextrin, D-fructose, D-fructose-6-PO_4_, D-fucose, D-galactose, D-galacturonic acid, D-gluconic acid, D-glucose-6-PO_4_, D-mannitol, D-mannose, D-sorbitol, formic acid, glucuronamide, L-fucose, L-galactonic acid lactone, L-histidine, L-lactic acid, L-rhamnose, myo-inositol, α-D-glucose and β-hydroxy-D,L-butyric acid as the sole carbon source, and amygdalin, arbutin, D-arabinose, D-arabitol, D-ardonitol, D-cellobiose, D-fructose, D-fucose, D-galactose, D-glucose, D-lyxose, D-mannitol, D-mannose, D-ribose, D-sorbitol, D-tagatose, D-trehalose, dulcitol, D-xylose, esculin ferric citrate, inositol, L-arabinose, L-fucose, L-rhamnose, L-sorbose, L-xylose, N-acetyl-glucosamine, potassium 2-ketogluconate, potassium 5-ketogluconate, salicin and xylitol can be assimilated to produce acid. Positive alkaline phosphatase, cystine arylamidase, esterase(C4), esterase lipase(C8), leucine arylamidase, naphthol-AS-B1-phosphohydrolase, valine arylamidase and β-glucuronidase, weakly positive for acid phosphatase and trypsin in API ZYM strip. The predominant polar lipids are diphosphatidylglycerol (DPG), phosphatidylglycerol (PG), phosphatidylcholine (PC), phosphatidylethanolamine (PE), an unidentified phospholipid (PL) and an unidentified aminolipid (AL). The sole respiratory quinone is Q-10. The major cellular fatty acids are C_18:1_ω7*c*/C_18:1_ω6*c*, cyclo-C_19:0_ω8*c*, C_16:0_ and C_18:0_. The genome sequence is characterized by a size of 6.02 Mbp and the G+C content of 67.3 %. The type strain CPCC 101083^T^ (=NBRC 113514^T^ =KCTC 62854^T^) was isolated from a sandy dune sample with moss-dominated crusts collected from Badain Jaran desert, China. The DDBJ/EMBL/GenBank accession numbers of 16S rRNA gene sequence and draft genome sequence of the strain CPCC 101083^T^ are MK392027 and JABGCL000000000, respectively.

The authors apologize for this error and state that this does not change the scientific conclusions of the article in any way.

