# Supplementary material for: Geminicoccus flavidas sp. nov. and Geminicoccus harenae sp. nov., two IAA-producing novel rare bacterial species inhabiting desert biological soil crusts
Source: Front Microbiol. 2022 Oct 31;13:1034816. doi: 10.3389/fmicb.2022.1034816 (PMC9659566; doi:10.3389/fmicb.2022.1034816)
Supplement: Supplementary file 1 [file Data_Sheet_1.pdf]

Geminicoccus flavidas sp. nov. and *Geminicoccus harenae* sp. nov., Two IAA-producing Novel Rare Bacterial Species Inhabiting Desert Biological Soil Crusts

Zhu-Ming Jiang[1,2], Yang Deng[1,2], Xue-Fei Han[1,2], Jing Su[1], Hao Wang[1], Li-Yan Yu[1], Yu-Qin Zhang[1,2*]

[1]Institute of Medicinal Biotechnology, Chinese Academy of Medical Sciences & Peking Union Medical College, Beijing 100050, P. R. China

[2]State Key Laboratory of Dao-di Herb, Beijing, 100700, P. R. China

[*]Author for correspondence:

Yu-Qin Zhang

Tel: +86-10-83167110

Fax: +86-10-83167110

E-Mail: yzhang@imb.pumc.edu.cn

Keywords: ***Geminicoccus flavidas*, *Geminicoccus harenae*, average nucleotide identity, pan-genome, biological soil crusts**

Abbreviation: DPG, diphosphatidylglycerol; PG, phosphatidylglycerol; PE, phosphatidylethanolamine; PC, phosphatidylcholine; PL, unidentified phospholipid; AL, unidentified aminophospholipid; ANI, average nucleotide identity; dDDH, digital DNA-DNA hybridization; IAA, indole-3-acetic acid.

FIGURE S1 | IAA standard curve and the absorbance values of strains CPCC 101082^T, CPCC 101083^T and DSM 18922^T in their respective fermentation broth at 540 nm.

Circles colored with orange, grey and yellow represent the strain CPCC 101082^T, CPCC 101083^T and DSM 18922^T, respectively.

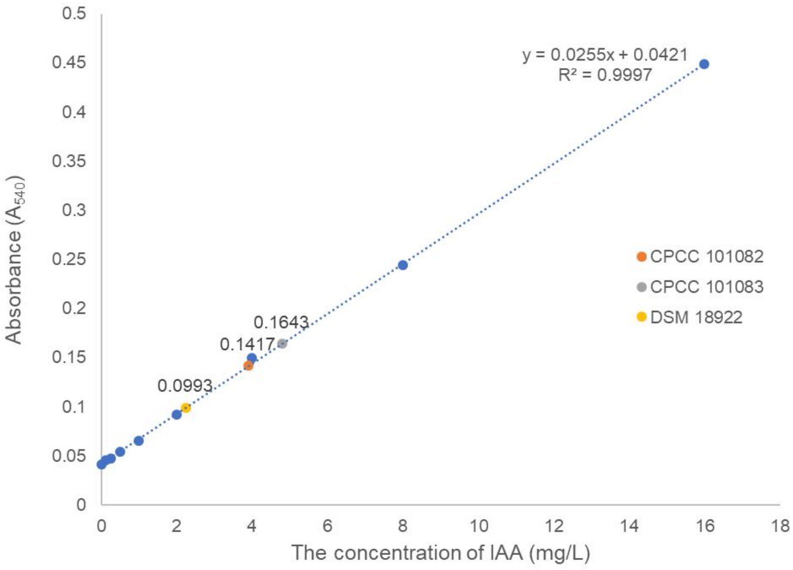

FIGURE S2 | Polar lipids profiles of the strain CPCC 101082^{T} and CPCC 101083^{T} separation by two-dimensional TLC.

(a) was detected by spraying with molybdatophosphoric acid reagent; (b) was detected by spraying with phosphate stain reagent; (c) was detected by spraying with ninhydrin stain reagent; (d) was detected by spraying with dragendorff reagent.

DPG, diphosphatidylglycerol; PG, phosphatidylglycerol; PC, phosphatidylcholine; PE, phosphatidylethanolamine; PL, unidentified phospholipid; AL, unidentified aminolipid; UL, unknown polar lipid.

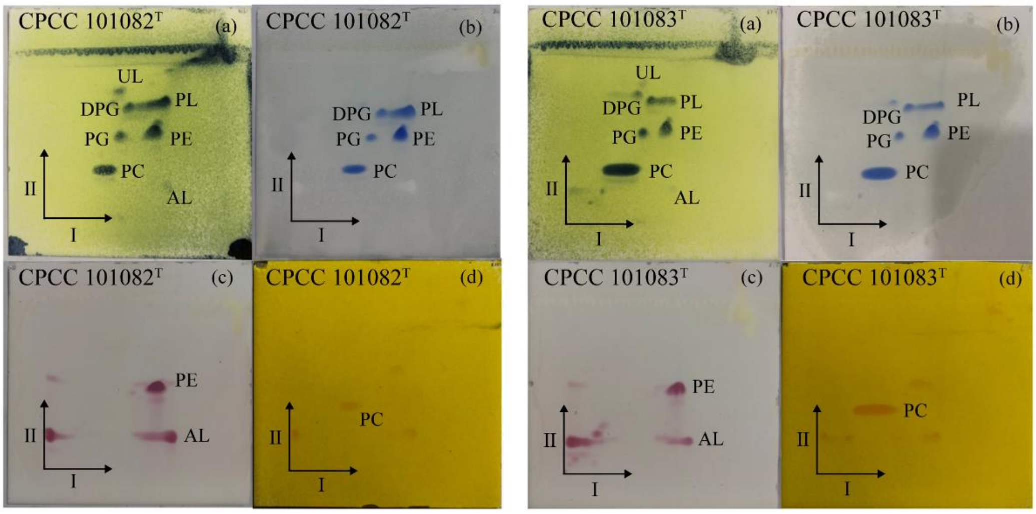

FIGURE S3 | Phylogenetic tree constructed by BPGA showing the relationship of the newly proposed species with other species of the family *Geminicoccaceae* based on concatenated core genes.

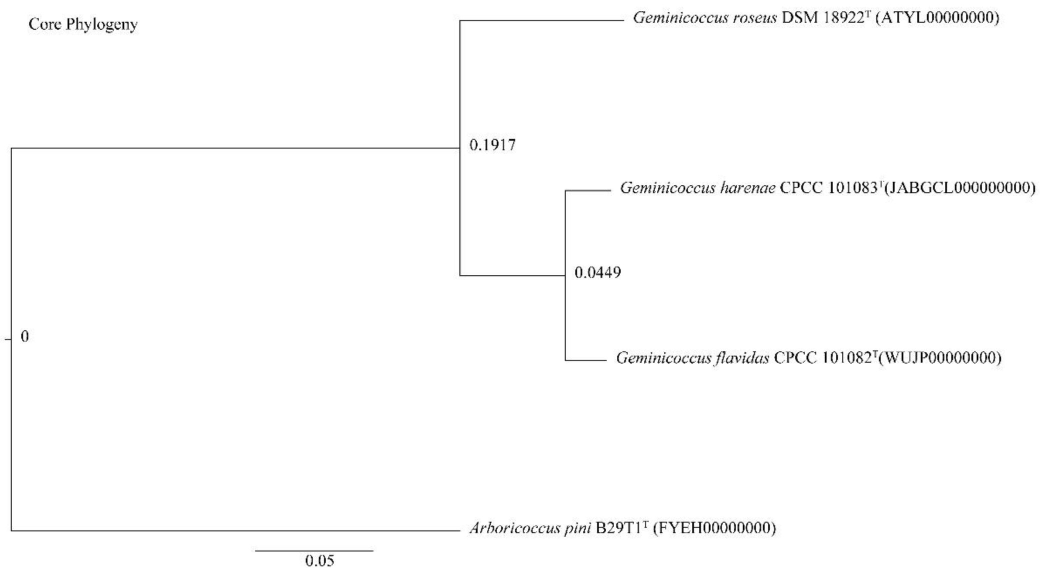

FIGURE S4 | Phylogenetic tree constructed by BPGA showing the relationship of the newly proposed species with other species of the family *Geminicoccaceae* based on binary gene presence/absence matrix (pan-matrix).

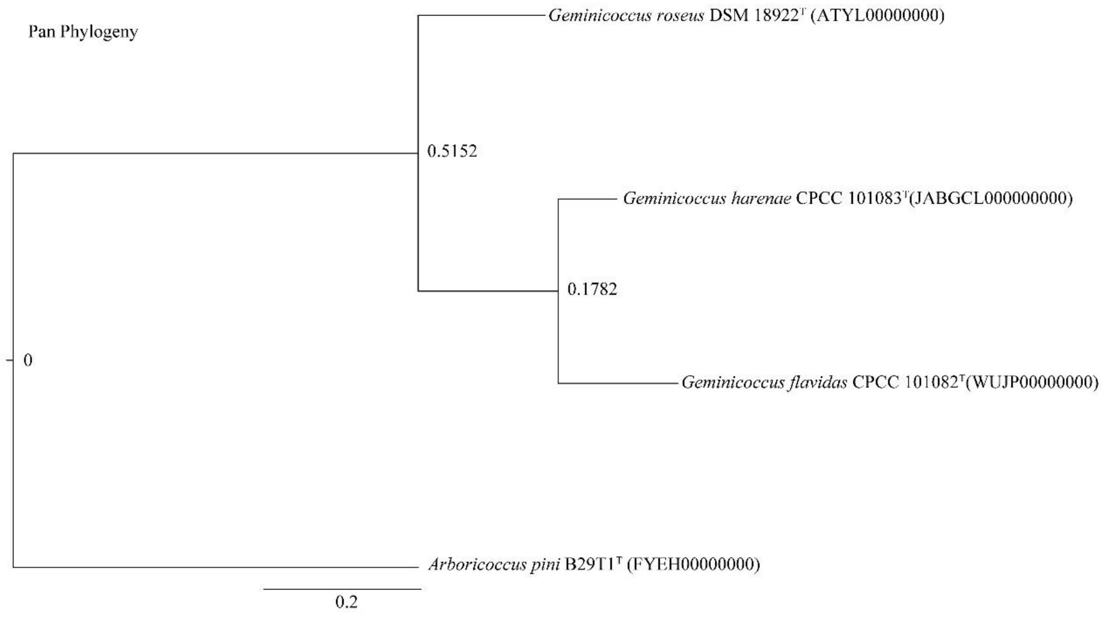

FIGURE S5 | Two KEGG pathways for autotrophic carbon dioxide fixation of CPCC 101082^T and CPCC 101083^T.

KEGG pathway diagram showing the genes of strain CPCC 101082^T and CPCC 101083^T encoding for enzymes in autotrophic carbon dioxide fixation pathways in prokaryotes other than the reductive pentose phosphate pathway (A and C). KEGG pathway diagram showing the genes of strains CPCC 101082^T and CPCC 101083^T encoding for enzymes in the reductive pentose phosphate pathway of autotrophic carbon dioxide fixation (B and D). The genes encoding for the enzymes present in the genome of CPCC 101082^T and CPCC 101083^T are in green.

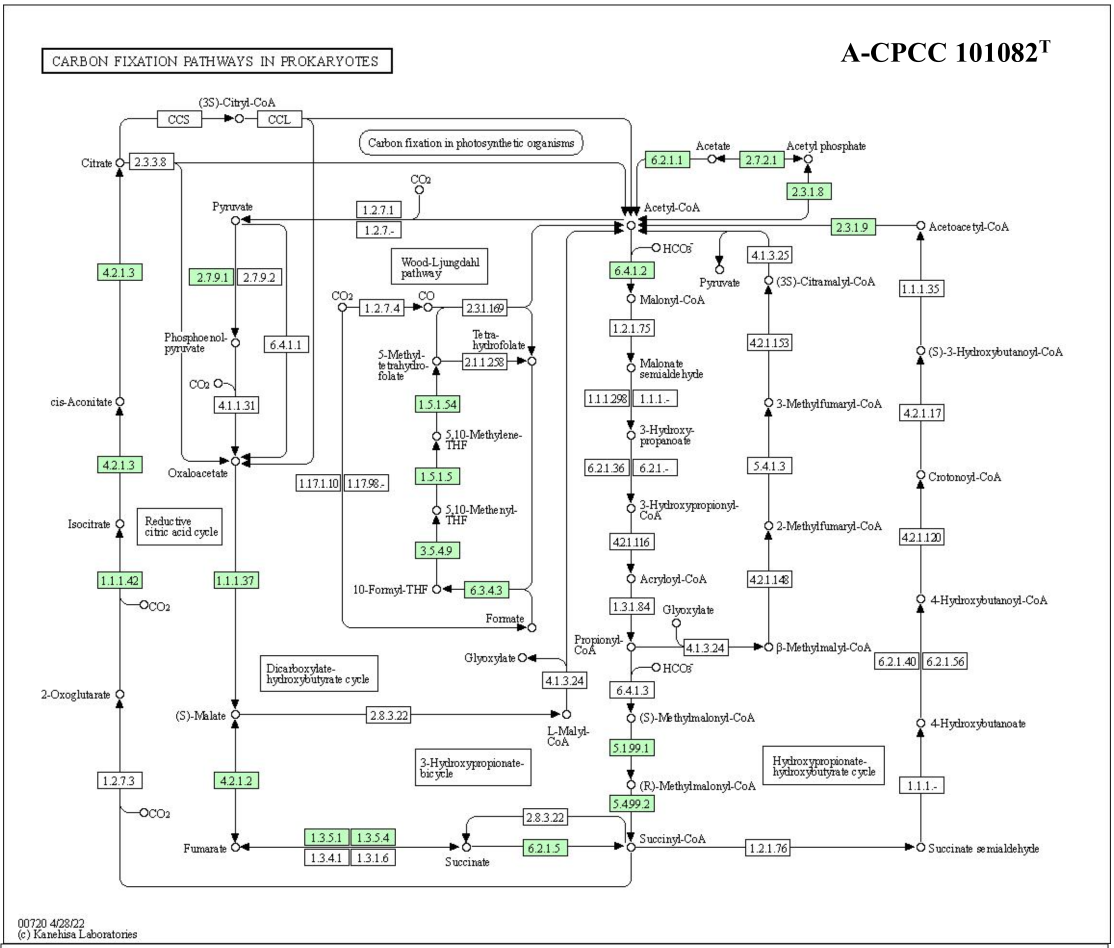
CARBON FIXATION PATHWAYS IN PROKARYOTES
A-CPCC 101082T
(3S)-Citryl-CoA
CCS
CCL
Citrate
2.3.3.8
Carbon fixation in photosynthetic organisms
Acetate
Acetyl phosphate
6.2.1.1
2.7.2.1
2.3.1.8
Pyruvate
CO2
1.2.7.1
1.2.7.-
Acetyl-CoA
2.3.1.9
Acetoacetyl-CoA
HCO3-
4.2.1.3
2.7.9.1
2.7.9.2
Wood-Ljungdahl pathway
6.4.1.2
Pyruvate
4.1.3.25
(3S)-Citramalyl-CoA
1.1.1.35
Malonyl-CoA
CO
1.2.7.4
2.3.1.169
Phosphoenol-pyruvate
6.4.1.1
5-Methyl-tetrahydro-folate
Tetra-hydrofolate
2.1.1.258
1.2.1.75
4.2.1.153
(S)-3-Hydroxybutanoyl-CoA
Malonate semialdehyde
cis-Aconitate
4.1.1.31
1.5.1.54
1.1.1.298
1.1.1.-
3-Methylfumaryl-CoA
4.2.1.17
5,10-Methylene-THF
3-Hydroxy-propanoate
4.2.1.3
Oxaloacetate
1.17.1.10
1.17.98.-
1.5.1.5
6.2.1.36
6.2.1.-
5.4.1.3
Crotonoyl-CoA
Isocitrate
Reductive citric acid cycle
5,10-Methenyl-THF
3-Hydroxypropionyl-CoA
2-Methylfumaryl-CoA
4.2.1.116
4.2.1.120
3.5.4.9
1.1.1.42
1.1.1.37
Acryloyl-CoA
4.2.1.148
4-Hydroxybutanoyl-CoA
10-Formyl-THF
6.3.4.3
1.3.1.84
Glyoxylate
Formate
Propionyl-CoA
4.1.3.24
β-Methylmalyl-CoA
Glyoxylate
Dicarboxylate-hydroxybutyrate cycle
HCO3-
6.2.1.40
6.2.1.56
4.1.3.24
2-Oxoglutarate
6.4.1.3
(S)-Malate
2.8.3.22
L-Malyl-CoA
(S)-Methylmalonyl-CoA
4-Hydroxybutanoate
3-Hydroxypropionate-bicycle
5.1.99.1
Hydroxypropionate-hydroxybutyrate cycle
1.2.7.3
4.2.1.2
(R)-Methylmalonyl-CoA
1.1.1.-
5.4.99.2
2.8.3.22
1.3.5.1
1.3.5.4
Succinyl-CoA
Fumarate
6.2.1.5
1.2.1.76
Succinate semialdehyde
1.3.4.1
1.3.1.6
Succinate
00720 4/28/22
(c) Kanehisa Laboratories

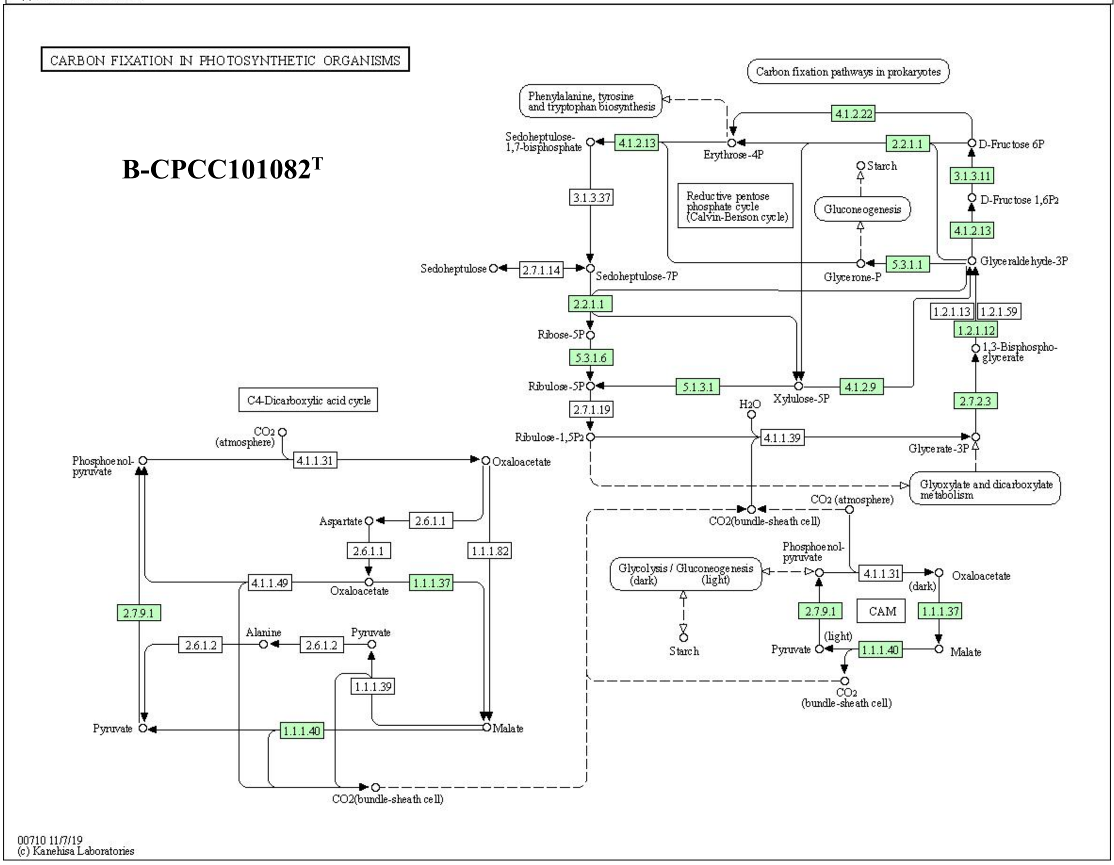
CARBON FIXATION IN PHOTOSYNTHETIC ORGANISMS
Carbon fixation pathways in prokaryotes
Phenylalanine, tyrosine and tryptophan biosynthesis
4.1.2.22
Sedoheptulose-1,7-bisphosphate
4.1.2.13
2.2.1.1
D-Fructose 6P
Erythrose-4P
B-CPCC101082T
Starch
3.1.3.11
3.1.3.37
Reductive pentose phosphate cycle (Calvin-Benson cycle)
D-Fructose 1,6P2
Gluconeogenesis
4.1.2.13
Sedoheptulose
2.7.1.14
Sedoheptulose-7P
5.3.1.1
Glyceraldehyde-3P
Glycerone-P
2.2.1.1
1.2.1.13
1.2.1.59
Ribose-5P
1.2.1.12
1,3-Bisphospho-glycerate
5.3.1.6
Ribulose-5P
5.1.3.1
4.1.2.9
Xylulose-5P
C4-Dicarboxylic acid cycle
2.7.1.19
H2O
2.7.2.3
CO2 (atmosphere)
Ribulose-1,5P2
4.1.1.39
Glycerate-3P
Phosphoenol-pyruvate
4.1.1.31
Oxaloacetate
Glyoxylate and dicarboxylate metabolism
CO2 (atmosphere)
CO2(bundle-sheath cell)
Aspartate
2.6.1.1
2.6.1.1
1.1.1.82
Phosphoenol-pyruvate
Glycolysis / Gluconeogenesis (dark) (light)
4.1.1.31
Oxaloacetate
(dark)
4.1.1.49
Oxaloacetate
1.1.1.37
2.7.9.1
2.7.9.1
CAM
1.1.1.37
Alanine
Pyruvate
(light)
2.6.1.2
2.6.1.2
Starch
Pyruvate
1.1.1.40
Malate
1.1.1.39
CO2 (bundle-sheath cell)
Pyruvate
1.1.1.40
Malate
CO2(bundle-sheath cell)
00710 11/7/19
(c) Kanehisa Laboratories

C- CPCC 101083ᵀ

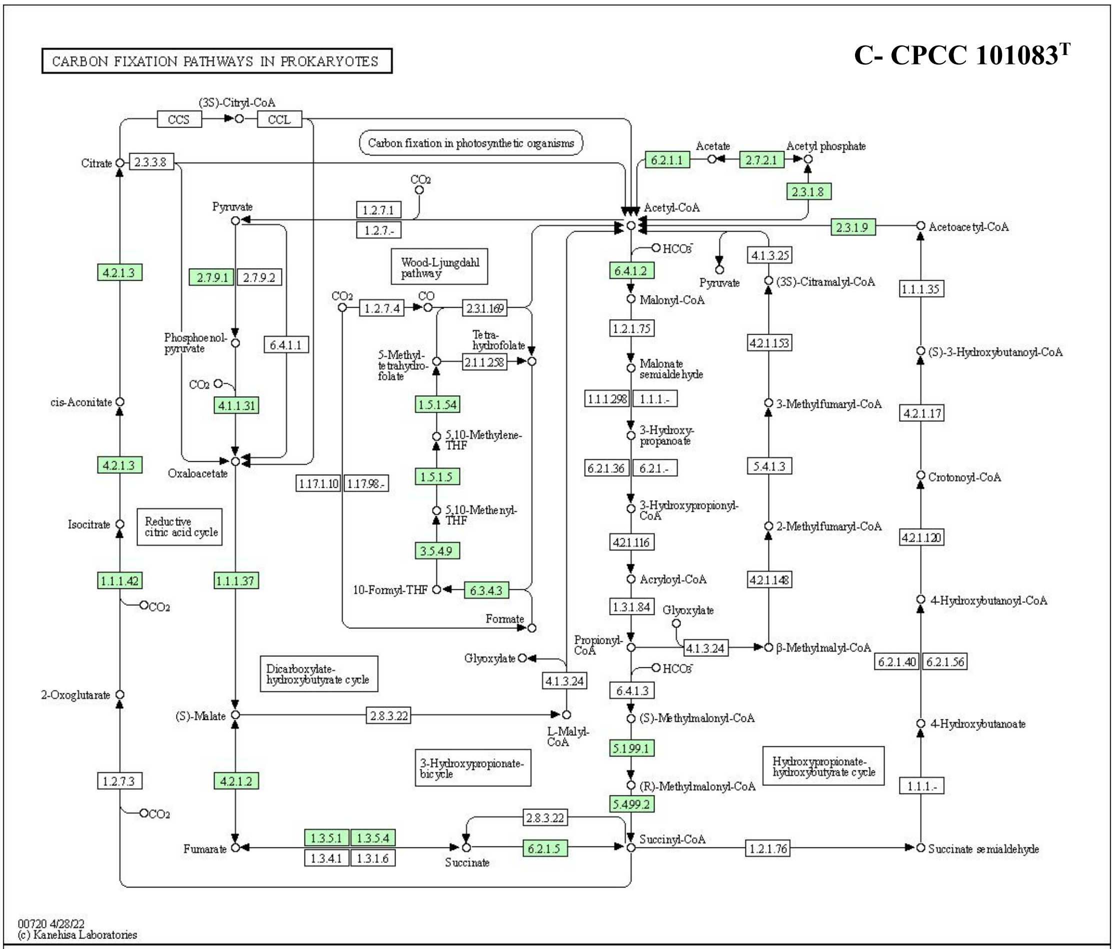

D-CPCC 101083ᵀ

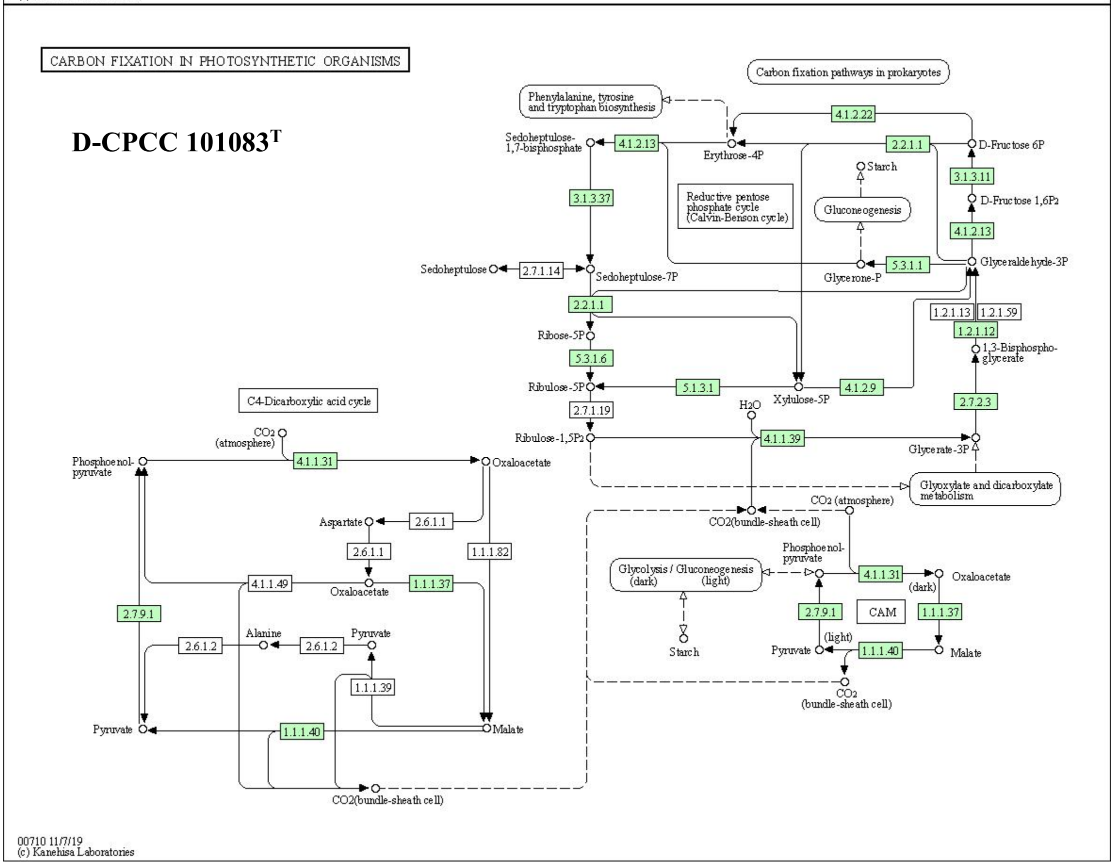

Table S1 Fatty acids profiles of strains CPCC 101082^{T}, CPCC 101083^{T} and *Geminicoccus roseus* DSM 18922^{T}.

TR, trace amount (<0.5 %); ND, not detected. Data of the species *Geminicoccus roseus* DSM 18922^{T} was adopted from Proença *et al.* Summed feature 2 contains $C_{12:0}$ aldehyde, $C_{14:0}$ 3-OH and/or iso-$C_{16:1}$; Summed feature 3 contains $C_{16:1}$ $\omega 7c$ and/or iso-$C_{15:0}$ 2-OH; Summed feature 4 contains iso-$C_{17:1}$ I and/or anteiso- $C_{17:1}$ B; Summed feature 8 contains $C_{18:1}$ $\omega 7c$ and/or $C_{18:1}$ $\omega 6c$.

Fatty acid (%)	CPCC 101082^{T}	CPCC 101083^{T}	DSM 18922^{T}
**Straight-chain fatty acid**			
$C_{14:0}$	TR	TR	ND
$C_{16:0}$	10.8	14.1	14.0±0.2
$C_{17:0}$	0.6	0.5	ND
$C_{18:0}$	1.8	8.9	1.8±0.1
**Hydroxy fatty acids**			
$C_{16:0}$ 2-OH	TR	ND	ND
$C_{16:0}$ 3-OH	1.8	0.9	1.8±0.0
$C_{18:0}$ 3-OH	TR	1.8	1.9±0.1
**0thers**			
Sum In Feature 2	TR	TR	1.1±0.0
Sum In Feature 3	2.1	0.8	2.3±0.0
Sum In Feature 4	2.2	0.6	ND
Sum In Feature 8	29.0	37.4	50.6±0.8
$C_{16:1}$ $\omega 5c$	0.8	TR	TR
$C_{17:1}$ $\omega 6c$	4.5	ND	ND
$C_{17:1}$ $\omega 8c$	0.5	ND	ND
$C_{18:1}$ $\omega 5c$	2.0	0.5	TR
$C_{18:1}$ $\omega 7c$ 11-methyl	TR	TR	3.3±0.2
cyclo-$C_{17:0}$	ND	ND	1.0±0.0
cyclo-$C_{19:0}$ $\omega 8c$	40.4	31.8	19.1±0.3

Table S2 The pairwise values of 16S rRNA gene similarity, dDDH and ANI among strains CPCC 101082^T, CPCC 101083^T and *Geminicoccus roseus* DSM 18922^T.

Note: ANI, values of average nucleotide identity; dDDH, digital DNA-DNA hybridization. The values in parentheses are 16S rRNA gene similarity, dDDH and ANI from left to right.

	G. roseus DSM 18922^T	CPCC 101083^T
CPCC 101082^T	(96.9, 23.0, 80.6)	(98.6, 47.0, 92.4)
CPCC 101083^T	(96.9, 23.6, 81.2)	/

Table S3 Secondary metabolite biosynthesis gene clusters predicted from the 3 strains' genomes of the genus *Geminicoccus*.

Genomic location	Type	Secondary metabolite synthesis gene cluster	Smilarity
**CPCC 101082^T**			
Scaffold2	NAGGN	Alkaloid	5 %
Scaffold5	NAPAA	/	/
Scaffold6	terpene	NRP: Cyclic depsipeptide + Polyketide: Modular type I	9 %
Scaffold7	T3PKS	oxalomycin B	6 %
Scaffold8	NRPS	bacillibactin NRP	23 %
Scaffold10	terpene	/	/
Scaffold20	thioamitides	/	/
Scaffold36	T1PKS	Ibomycin (Polyketide)	12%
Scaffold49	RRE-containing	/	/
Scaffold88	T1PKS	/	/
**CPCC 101083^T**			
Scaffold7	terpene	NRP: Cyclic depsipeptide + Polyketide: Modular type I	9 %
Scaffold11	T3PKS	oxalomycin B (NRP+Polyketide)	6 %
Scaffold14	NRPS	bacillibactin (NRP)	15 %
Scaffold17	NAPAA	/	/
Scaffold20	thioamitides	/	/
Scaffold23	NAGGN	/	/
Scaffold31	terpene	/	/

Scaffold39	T1PKS	Polyketide	32%
Scaffold52	RRE-containing	/	/
Scaffold138	T1PKS	/	/
**DSM 18922^T**			
NZ_KE386572.1	NRPS/ T1PKS	ajudazol A (NRP: + Polyketide: Modular type I)	30 %
NZ_KE386572.1	terpene	/	/
NZ_KE386572.1	NAGGN	/	/
NZ_KE386572.1	redox-cofactor	paulomycin	3 %
NZ_KE386572.1	terpene	NRP: Cyclic depsipeptide + Polyketide: Modular type I	9 %
NZ_KE386572.1	thioamitides	/	/
NZ_KE386572.1	RiPP-like	Colicin V (RiPP)	1%
